# Dynamic functional connectivity between nucleus accumbens and the central executive network relates to chronic cannabis use

**DOI:** 10.1002/hbm.25036

**Published:** 2020-05-20

**Authors:** Hye Bin Yoo, Blake Edward Moya, Francesca M. Filbey

**Affiliations:** ^1^ Center for BrainHealth, School of Behavioral and Brain Sciences University of Texas at Dallas TX USA; ^2^ Department of Neurological Surgery University of Texas Southwestern Dallas TX USA

**Keywords:** cannabis, central executive network, cue exposure task, dynamic connectivity, nucleus accumbens

## Abstract

The neural mechanisms of drug cue‐reactivity regarding the temporal fluctuations of functional connectivity, namely the dynamic connectivity, are sparsely studied. Quantifying the task‐modulated variability in dynamic functional connectivity at cue exposure can aid the understanding. We analyzed changes in dynamic connectivity in 54 adult cannabis users and 90 controls during a cannabis cue exposure task. The variability was measured as standard deviation in the (a) connectivity weights of the default mode, the central executive, and the salience networks and two reward loci (amygdalae and nuclei accumbens); and (b) topological indexes of the whole brain (global efficiency, modularity and network resilience). These were compared for the main effects of task conditions and the group (users vs. controls), and correlated with pre‐ and during‐scan subjective craving. The variability of connectivity weights between the central executive network and nuclei accumbens was increased in users throughout the cue exposure task, and, was positively correlated with during‐scan craving for cannabis. The variability of modularity was not different by groups, but positively correlated with prescan craving. The variability of dynamic connectivity during cannabis cue exposure task between the central executive network and the nuclei accumbens, and, the level of modularity, seem to relate to the neural underpinning of cannabis use and the subjective craving.

## 1 INTRODUCTION

Craving is a hallmark behavioral problem in substance use disorders (SUDs) that contributes toward loss of control over substance use and drug relapse. One of its well‐known neural correlates is the reward‐related prefrontal‐limbic pathway, but the large‐scale intrinsic networks are also crucially involved (Filbey, Gohel, Prashad, & Biswal, 2018; Zilverstand, Huang, Alia‐Klein, & Goldstein, 2018). Some of those networks are the default mode (DMN), the central executive (CEN), and the salience networks (SN). The DMN, or self‐oriented network, relates to attention toward internal information (Raichle, 2015). Its activity correlates with the subjective craving, and seems to show reduced functional interactions with the other brain networks in SUDs (Zhang & Volkow, 2019). The CEN, or goal‐oriented network, relates to executive and inhibitory controls that drive attention on external objectives, and control impulsivity (Chen et al., 2013; Sridharan, Levitin, & Menon, 2008), functions that are often compromised in SUDs (Dalley & Robbins, 2017; Luijten et al., 2014). The SN is implicated in allocating functional resources between the DMN and CEN based on the salience of information (Menon & Uddin, 2010; Seeley et al., 2007; Sridharan et al., 2008). Its dysfunction potentially affects the attentional attribution to external information, which relates to an imbalance of attention to drug versus nondrug cues (R. Z. Goldstein & Volkow, 2011; Naqvi & Bechara, 2009; Sutherland, McHugh, Pariyadath, & Stein, 2012).

The functional underpinning of bias toward drug‐related information have been found in subcortical regions in prefrontal‐limbic pathway (Filbey & Dunlop, 2014), such as the nucleus accumbens and amygdala (Swanson, 1982). Both nucleus accumbens and amygdala receive dopamine (DA) inputs within the mesocorticolimbic pathway, and are important in acquiring motor behavior under conditioning toward drug cues (Pert, Post, & Weiss, 1990). Specifically, the nucleus accumbens (NAcc), also referred to as ventral striatum, plays a key role in appraising the subjective value of reward (Milton & Everitt, 2012) toward reward‐motivated decision making (Cools, 2015). The amygdala (Amyg) seems to appraise the emotional relevance of external information related to reward (Baxter & Murray, 2002; Cunningham & Brosch, 2012). The DA system can influence the functional connectivity in DMN, CEN, SN (Cole, Oei, et al., 2013) as well as NAcc and Amyg, which may also be associated with the reduced inhibitory control and biased salience attribution found in SUDs (R. Z. Goldstein & Volkow, 2011; Zilverstand et al., 2018).

Most of the previous studies on the task‐modulated functional connectivity (FC) during cue‐induced craving applied static approaches, which do not take the variability of FC over time into account. One of the most widely utilized methods for analyzing task‐modulated static FC is psychophysiological interaction (PPI; Friston et al., 1997; McLaren, Ries, Xu, & Johnson, 2012), which evaluates the correlations of task‐modulated blood‐oxygen‐level‐dependent (BOLD) response of one upon the other regions across the entire observation time. However, high‐frequency phasic activity of DA neurons in response to drug cues (Koob & Volkow, 2010) may manifest temporal fluctuations in FC. The variability can be quantified using dynamic FC, which reflects time‐dependent changes of FC by subdividing the entire observation into smaller sections instead of across all frames within the observation time (Allen et al., 2012; Chang & Glover, 2010; Preti, Bolton, & van de Ville, 2017; Sakoglu et al., 2010; Sakoglu & Calhoun, 2009a, 2009b; Sakoglu, Michael, & Calhoun, 2009). FC of the brain and its topological properties are inherently nonstationary (the connectivity mean and variability are not constant over observation time) depending on the given tasks (Braun et al., 2015; Fong et al., 2019; Gonzalez‐Castillo & Bandettini, 2018) and also during the resting state (Allen et al., 2012; Chang & Glover, 2010; de Pasquale, Della Penna, Sporns, Romani, & Corbetta, 2016; Preti et al., 2017). Dynamic FC can capture the time‐dependent changes that relate to temporally dynamic processes, for example, phasic neural firing of DA neurons.

Dynamic conditional correlation (DCC) quantifies the temporally sensitive alterations in functional coherence across brain regions per frame (Engle, 2002; Lindquist, Xu, Nebel, & Caffo, 2014). Based on framewise changes in FC, DCC can account for model‐free, momentary FC changes in a specific task condition, therefore expanding from the model‐based estimation of FC via generalized PPI. By temporally weighting the DCC by task conditions, we can obtain the series of dynamic FC when a task condition was in effect and the task‐modulated variability of dynamic FC in standard deviation (*SD*).

Previous studies on the alterations of dynamic FC related to substance use are sparse. Vergara, Weiland, Hutchison, and Calhoun (2018) combined large number of users dependent on various kinds of substances and found that the dwelling times of specific functional states represented by dynamic FC patterns differ significantly across user groups of substances including cannabis. Focusing on cannabis use, Zaytseva et al. (2019) found a specific dynamic FC pattern that shows higher connectivity within and between auditory and somatomotor cortices, which appears during acute delta‐9‐tetrahydrocannabiol (THC) intoxication in occasional users. Sakoglu et al. (2019) is one of the earlier examples that compared dynamic FC patterns using independent component analysis during motor response inhibition task. This study noted pairs of networks that differentiate cocaine users from the healthy controls, which include visual, sensorimotor, the DMN and the CEN. In sum, there is emerging literature demonstrating that dynamic FC is a sensitive marker for substance use and related behaviors. Dynamic FC may also clarify the functional states precipitated by drug‐related conditions. This study therefore aimed to explore this possibility, by quantifying the dynamic FC changes directly relevant to the subjective craving, targeting the task‐modulated dynamic FC in the cannabis cue exposure task.

This paper investigated the dynamic FC during cannabis cue‐reactivity using DCC. We aimed to (a) identify a system‐level marker related to cue‐induced changes in the DMN, the CEN, and the SN, and Amyg and NAcc; (b) identify the difference in DCC‐derived dynamic FC measures across task conditions, and between cannabis users and controls; and (c) test whether these measures are associated with subjective craving.

## 2 MATERIALS AND METHODS

### 2.1 Participants

Participants were recruited from a study of neural mechanisms related to cannabis craving described in Filbey et al. (2014) and Filbey et al. (2016). The study's inclusion criteria were right‐handedness, English as the primary language, the absence of either a current psychosis, history of psychosis, traumatic brain injury, or MRI contraindications (e.g., pregnancy, MR‐incompatible metallic implants, claustrophobia, etc.). The nonusing controls (CON) were recruited based on the absence of daily cannabis use at any period in their lifetime, as well as an absence of current illicit drug use in the past 60 days. Chronic cannabis users (CAN) were defined as those with self‐reported history of regular cannabis use resulting in a minimum of 5,000 separate lifetime occasions of use. An additional inclusion criterion for CAN was self‐reported daily cannabis use over the preceding 60 days as determined by the Timeline Followback Calendar (TLFB). All the participants were excluded if drugs other than cannabis were detected by a urinary toxicity test, unless it was a prescribed medication. Participants with significant levels of other substance use as indicated by the Structured Clinical Interview for DSM‐IV criteria (First & Gibbon, 2004) were excluded from the study. Given the high comorbidity of tobacco and cannabis use, we did not exclude participants with regular tobacco use.

All participants were asked to refrain from alcohol use for 24 hr, and caffeine and cigarettes for the 2 hr before the scheduled scan. In addition, cannabis using participants were asked to abstain from using cannabis for 72 hr. Those whose self‐report did not meet this requirement were excluded from the study. As a result, a total of 144 participants (90 controls in CON group, 54 users in CAN group) were included in the study. Demographic information is shown in Table 1.

**TABLE 1:** Demographics of CON and CAN groups

	CON (*n* = 90)	CAN (*n* = 54)	Difference
Sex (*N*)	Male (*n* = 45)	Male (*n* = 31)	χ^2^ = 0.743
Age	29.40 ± 10.15	29.35 ± 7.93	*t*(132.237) = 0.032
Cigarette use per day^a^	1.92 ± 4.13	5.11 ± 8.29	*t*(37.894) = −1.623
Cannabis use variables			
Onset age of regular use^b^	N/A	18.50 ± 3.80 years	N/A
Duration of regular use^c^	N/A	10.26 ± 7.56 years	N/A
Last cannabis use before scan^d^	N/A	79.43 ± 7.34 hr	N/A
Cannabis grams per day^e^	N/A	2.12 ± 1.69 g	N/A
THC/creatinine ratio^f^	N/A	2.18 ± 1.90 ng/ml	N/A
Craving before scan^g^	N/A	253.88 ± 160.76 points	N/A

*Note:* Values are shown in mean ± standard deviation unless specified. Group differences show results from chi‐square test, or independent *t*‐test with equal variances not assumed.

a This value refers to the answer for “Since you started regular daily smoking, what is the average number of cigarettes you smoked per day?” (13 CON participants, 27 CAN participants applied).

b Substance History Questionnaire for marijuana use #2, “The age one has started using cannabis regularly,” only accounting for group CAN (*n* = 54).

c Substance History Questionnaire for marijuana use #3, “The number of years one has been using cannabis regularly” (*n* = 54).

d The self‐reported date of last cannabis use before the scan (*n* = 43).

e The self‐reported level of cannabis intake every day (*n* = 54).

f THC metabolites over creatinine (ng/ml) measured from urine before MR scan (*n* = 53).

g Subjective level of craving before the cue task in the scanner, based on the summed score from Marijuana Craving Questionnaire (*n* = 52).

### 2.2 Cannabis use measures

We collected data related to age of first regular use, and the duration of regular use of cannabis using the Substance History Questionnaire (Sobell, Kwan, & Sobell, 1995). Self‐reports of the last date of cannabis use before the scan date, and the level of cannabis intake were evaluated in grams per day. Urine THC (ng) over creatinine (ml) levels were measured via gas chromatography/mass spectrometry.

### 2.3 Subjective craving measures

Subjective craving for cannabis was measured according to the following: (a) prescan/baseline subjective craving—assessed using the total score from the Marijuana Craving Questionnaire (Haughey, Marshall, Schacht, Louis, & Hutchison, 2008) prior to the fMRI scan; (b) during‐scan/cue‐induced subjective craving—response on a 10‐point Likert scale following each trial of *cannabis* cue exposure in the fMRI (see details in task description below). Prescan, and the average of during‐scan scores were normalized using the mean and the *SD* of each within the CAN group (*n* = 52 for prescan craving, and *n* = 54 for during‐scan craving).

### 2.4 MR acquisition

All the MRI images were collected using a 3T Philips whole body scanner with the Quasar gradient subsystem (40 mT/m amplitude, a slew rate of 220 mT/m/ms) at Advanced Imaging Research Center at University of Texas Southwestern Medical Center. Structural T1 images were acquired in an MPRAGE sequence with the parameters: TR/TE = 8.1/3.7 ms, FA = 12 deg, voxel = 1 × 1 × 1 mm^3^, matrix size in *x* and *y* directions = 256 × 256, and FOV = 256 × 256 mm^2^.

Task‐based functional MRI (fMRI) was collected using a gradient echo, echo‐planar sequence. Parameters were: 810 dynamic scans (27 min), TR/TE = 2000/29 ms, FA = 75 deg, voxel = 3.44 × 3.44 × 3.50 mm^3^, matrix size in x and y directions = 64 × 64 mm^2^, and FOV = 220 × 220 mm^2^.

### 2.5 Cannabis cue exposure task

This study used a cue exposure task originally described in Filbey et al. (2016), which was modified from Filbey, Schacht, Myers, Chavez, and Hutchison (2009). The task consisted of two consecutive sessions that are 13 min and 30 s long, each one with a pseudorandom order of visual and tactile presentations of (a) a single cannabis cue, (b) a single natural reward cue, and (c) a single neutral cue. Each type of cue was presented for 12 trials, and for a single trial a cue was shown for 20 s long. Right after exposure, the subjective craving was quantified by asking participants to respond to: “Please rate your urge to use marijuana right now.” Responses were measured using a scale from 0 (no urge at all) to 10 (the highest) for 5 s, and the scores were recorded using a fiber‐optic pad. Participants who showed abnormally high levels of craving, which means the average rating score of higher than nine for all cues; and low levels of craving, which means the average rating score of zero for cannabis cues, were excluded from this study. During‐scan craving referred to the average of 12 rated scores after *cannabis* cues were presented.

**FIGURE 1:**
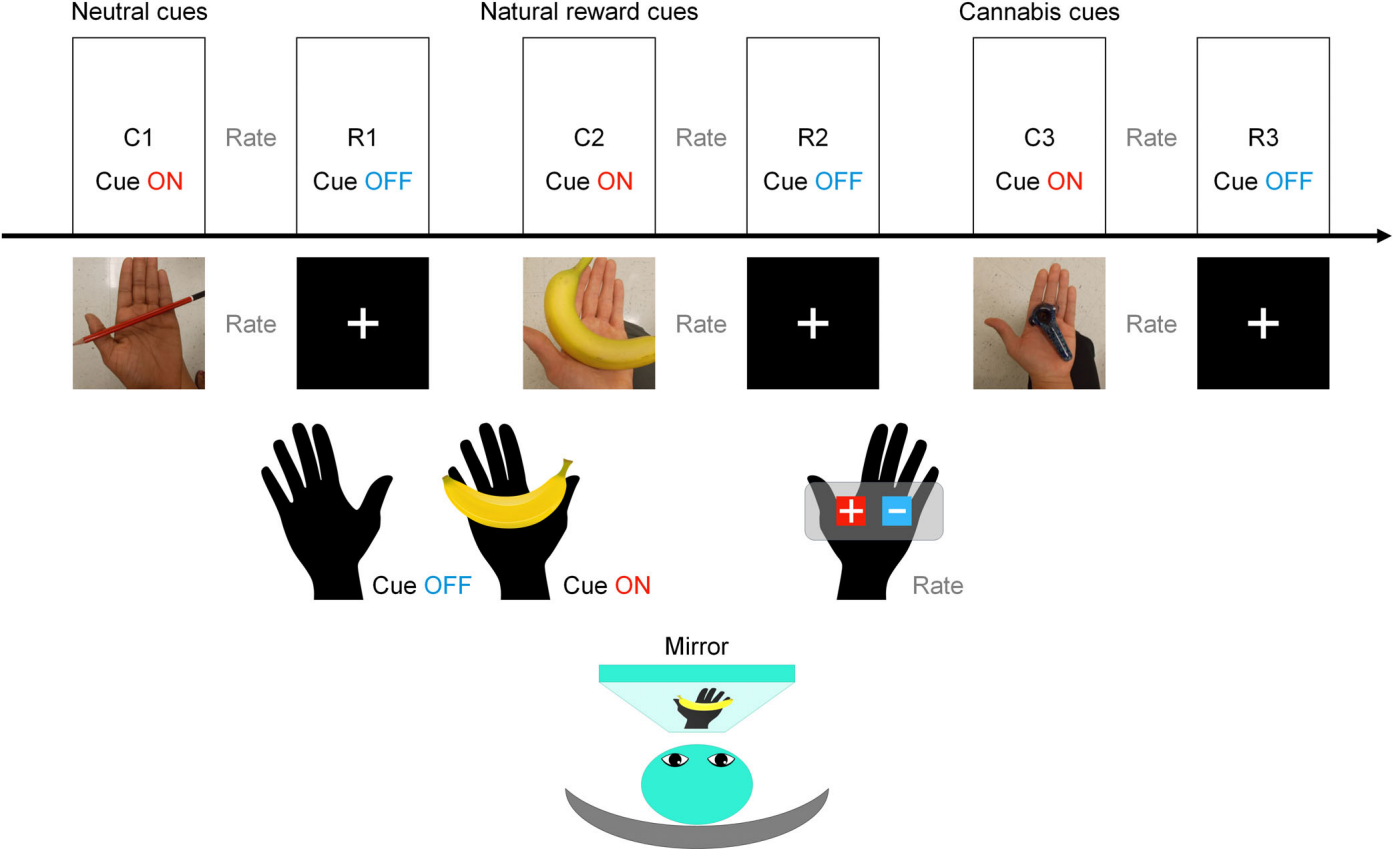
Illustration of cue exposure task

Cue stimuli given to the participants were based on their individual responses to “What is your preferred cannabis use method?” The answers were among a pipe, a bong, a blunt, and a joint. The same cues were randomly chosen for the controls. Natural reward cues were chosen as fruit (Filbey et al., 2016). Like the cannabis cues, we presented participants with their self‐selected fruit stimulus based on their responses to “what is your preferred fruit?” For both users and controls, the answers were among a banana, an apple, an orange, and grapes. Neutral cues were pencils for the participants in both groups. E‐Prime (Psychology Software Tools, Inc. E‐Prime 2.0. Retrieved from https://www.pstnet.com) was used to present cue and save the responses from the scanner. The timestamps of task responses were recorded in correspondence to the trigger pulses obtained from the scanner's magnet. Figure 1 illustrates the procedure of the cue exposure task.

In the following sections, FC measures were calculated in correspondence to each of six task conditions in abbreviations. C1 and W1 will be representing the conditions where neutral cues were presented (C1, meaning neutral cue ON state) and removed (W1, neutral cue OFF). C2 and W2 will be for natural reward cues (C2, natural reward cue ON and W2, natural reward cue OFF). C3 and W3 will be for cannabis cues (C3, cannabis cue ON and W3, cannabis cue OFF). The rated scores on the craving for cannabis cues in users’ group were used for further analyses. The subjective craving score was rated in both groups, but the post hoc analyses relating craving to FC measures were performed in users’ group only.

### 2.6 FMRI preprocessing

The preprocessing for fMRI was performed using the combination of fMRIPrep 1.2.5 (Esteban et al., 2019) that utilizes Nipype 1.1.6 and Nilearn 0.5.0 (https://nilearn.github.io/; K. Gorgolewski et al., 2011; K. J. Gorgolewski et al., 2017; Pedregosa et al., 2011), and CONN (Whitfield‐Gabrieli & Nieto‐Castanon, 2012). Basic preprocessing including framewise motion correction, extraction of motion‐related and physiological noise components, and, intermodal normalization of T1 and fMRI images onto the standard MNI space was performed using fMRIPrep. The pipeline of fMRIPrep utilized toolboxes from FMRIB Software Library (Jenkinson, Beckmann, Behrens, Woolrich, & Smith, 2012), FreeSurfer (Dale, Fischl, & Sereno, 1999; Fischl, Sereno, & Dale, 1999) and Advanced Normalization Tools (ANTs; Avants, Tustison, & Song, 2009). Further denoising of fMRI images using motion and physiological components, and, the integration of the fMRI images with the time series of the cue task were processed within a CONN pipeline.

The following descriptions on the preprocessing pipeline are adopted and revised from the fMRIPrep pipeline. A reference volume of an fMRI image was selected and brain‐extracted, then was linearly coregistered with nine degrees of freedom to the T1 images using BBRegister (FreeSurfer) that implements boundary‐based registration (Esteban et al., 2019; Greve & Fischl, 2009). Head motion across time per subject was estimated using MCFLIRT in FSL (Jenkinson, Bannister, Brady, & Smith, 2002), then the fMRI images were resampled to surfaces on fsaverage5 from FreeSurfer. The slice‐timing correction was applied, and the fMRI images were resampled back to the native space by applying a single, composite transform to correct for head‐motion and susceptibility distortions. Volumetric resamplings were performed using ANTs (Avants et al., 2009), configured with Lanczos interpolation to minimize the smoothing effects of other kernels (Lanczos, 1964), and surface resamplings were performed using FreeSurfer. The images were normalized to nonlinear and asymmetric MNI152 2009c standard space. Several confounding regressors were calculated, including framewise displacement, global signal, white matter, and cerebrospinal fluid signals (Power et al., 2014).

Further denoising was applied upon motion‐corrected and normalized fMRI images using motion‐related and physiological noise parameters estimated in the preliminary steps. A total of 36 confounding regressors, 24 of which are related to motion and 12 to physiological signals were linearly regressed out (Satterthwaite et al., 2013). For motion‐related regressors, first six were mean‐centered rotation and transition, second six were their mean‐centered derivatives, third six were their mean‐centered squared terms, and fourth six were their derivatives’ mean‐centered squared terms. For physiological signals, the same scheme was applied for three original signals, which were defined in global, white matter, and cerebrospinal fluid regions. The outlier timeframes that show exceptionally higher framewise displacement (larger than 0.5 mm) were linearly regressed out using binary vectors that label the bad frames (Power et al., 2014). Finally, the effects of cue exposure (C1, C2, C3), washout (W1, W2, W3) and subjective craving rating for each cue type (neutral, natural reward, cannabis cues) were linearly regressed out for each individual fMRI images, by accounting for the mean‐centered time series of the task convolved with canonical hemodynamic response function (HRF) and its derivative. Thus, a total of 18 (from three task conditions that are cue ON, cue OFF and rate, for three cue types, and both the original time series and its derivative = 3 × 3 × 2) regressors were additionally considered as noise components simultaneously with the other nuisance covariates. Rating (five‐second long) was not included in the assessment of task‐modulated connectivity, but its time series was used as a nuisance covariate. During the denoising step, the bandpass filtering was applied simultaneously with the other noise components, at low 0.01 to high 0.25 Hz.

### 2.7 Definition of brain regions and connections

FC was based on the cortical regions defined in the Gordon 333 atlas (Gordon et al., 2016), and 14 subcortical regions from Harvard‐Oxford atlas (Desikan et al., 2006; Frazier et al., 2005; J. M. Goldstein et al., 2007; Makris et al., 2006). Among a total 347 regions, 41 regions were in the default mode network (DMN), 24 regions in the central executive network (CEN), and 44 regions in the salience network (SN) that is labeled as either salience or cingulo‐opercular network (S. Sadaghiani & D'Esposito, 2015; Seeley et al., 2007) in the Gordon atlas. These three intrinsic networks of interest were accounted for the FC, as illustrated in Figure S1. In addition, this study considered two subcortical regions known to be important in SUDs: bilateral amygdalae (Amyg) and nuclei accumbens (NAcc).

A total of 12 functional connections were calculated using the time series per cortical region defined for each network, and subcortical regions of amygdala and nucleus accumbens. Specifically, these included (a) connections *within* each network that involve a pair of two regions from the same network (WithinDMN, WithinCEN, WithinSN); (b) connections *between* two networks that involve a pair of two regions from different networks (DMN–CEN, DMN–SN, CEN–SN); (c) connections between left and right amygdalae (Amyg) and each of three intrinsic networks (Amyg‐DMN, Amyg‐CEN, Amyg‐SN); and (4) connections between left and right nuclei accumbens (NAcc) and three networks (NAcc‐DMN, NAcc‐CEN, NAcc‐SN). Connection weights were averaged across all the pairs included per type of FC (e.g., for within the DMN, 41 × 40/2 = 820 connections’ weights were averaged) to represent each type of functional connectivity.

### 2.8 Dynamic functional connectivity via dynamic conditional correlation

Dynamic FC is a derivative measure of the FC that takes temporally dynamic changes in connectivity into account. Its quantification can be performed by either segregating the time series of BOLD activity into multiple chunks of smaller windows as in sliding‐window, or tapered sliding‐window approach (Allen et al., 2012; Chang & Glover, 2010; Sakoglu et al., 2009, 2010; Sakoglu & Calhoun, 2009a, 2009b), or estimating instantaneous FC without the need of defining the criteria for subdividing time series as in instantaneous phase coherence, multiple temporal derivative, or dynamic conditional correlation approach (Glerean, Salmi, Lahnakoski, Jääskeläinen, & Sams, 2012; Lindquist et al., 2014; Shine et al., 2015).

This study used dynamic conditional correlation (DCC) without moving average (Engle, 2002; Lindquist et al., 2014), which is based on the multivariate generalized autoregressive conditional heteroscedasticity model (Engle, 2002) that can be effective for estimating nonstationary temporal associations when the model of time series is well‐known (Lebo & Box‐Steffensmeier, 2008). We used the code implemented by Lindquist et al. (2014) shared in https://github.com/canlab/Lindquist_Dynamic_Correlation, which ran on MATLAB R2018b utilizing 120 high‐performance computing SLURM nodes. It first performs estimation of conditional variance of the two brain regions’ time series accounting for each present timeframe (*t*) and the past frames (*t* – 1, *t* – 2 … 1), then provides instantaneous DCC based on the variance values (Engle, 2002; Lindquist et al., 2014). The performance of DCC in estimating the ground‐truth dynamic connectivity is higher than the most of the sliding‐window approaches, given that the underlying parametric model for estimating variance is feasible (Lindquist et al., 2014). In comparisons across multiple datasets, the test–retest reliability was also higher for the DCC method (Choe et al., 2017). Applying moving average after obtaining DCC can improve its correspondence to behaviorally meaningful information during tasks (Xie et al., 2019), with the penalty of losing part of the information within the window and especially, at the edge of the very first or last series of windows. Leonardi and van de Ville (2015) provided mathematically feasible range of window length for moving average (window length ≥ 1/*f*_min_), but this study used DCC without moving average to retain as much information as possible throughout the entire scan. The following DCC metrics were calculated as our measure of dynamic FC.

#### 2.8.1 Primary DCC measures: Functional connectivity weights

**FIGURE 2:**
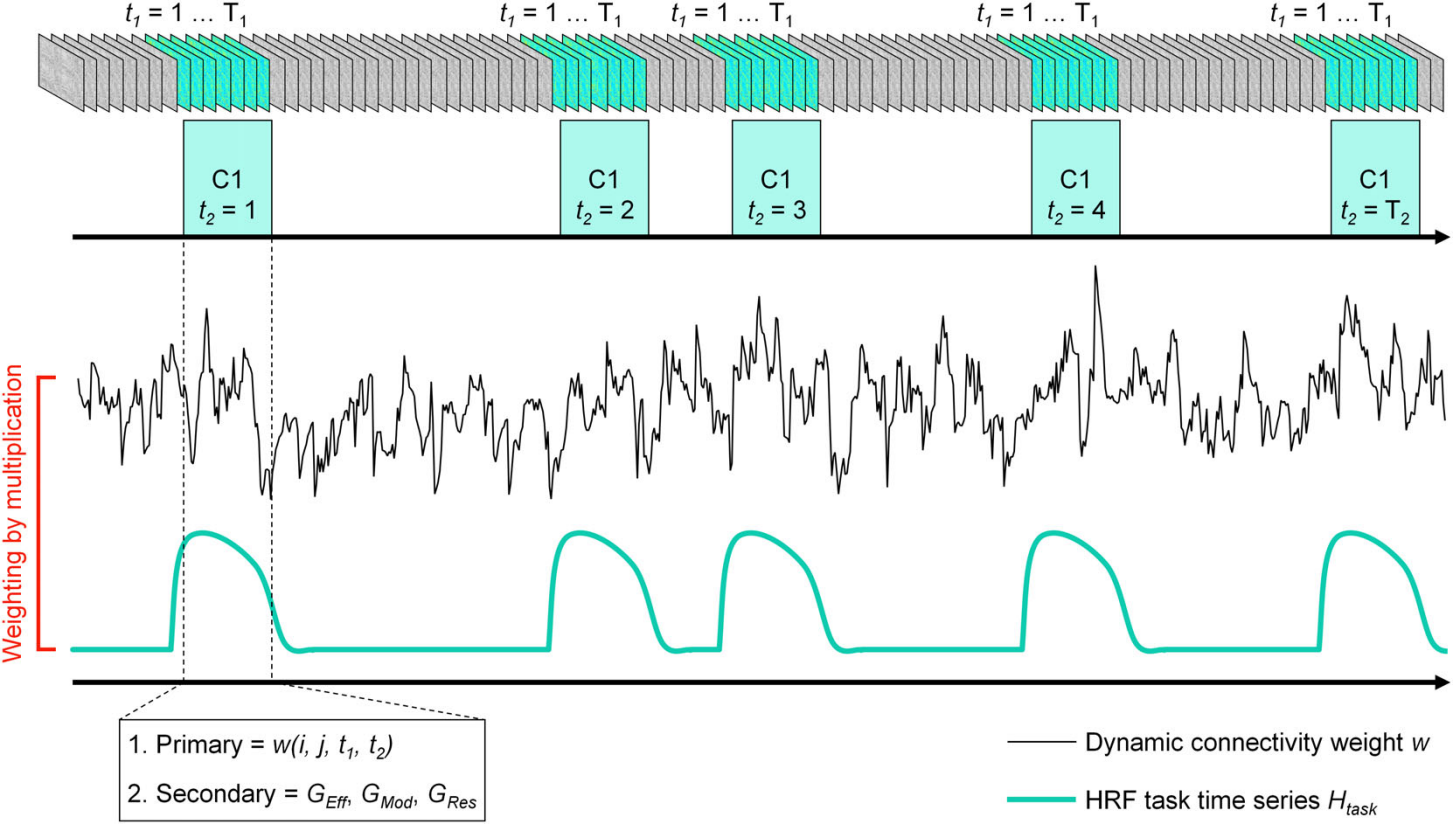
Illustration of primary and secondary dynamic functional connectivity measures defined per timeframe. The presentation is conceptual and not based on the actual data

The time series of DCC (347 ROIs × 347 ROIs × 810 frames) was calculated per individual. Correspondingly, the same 12 types of FC, which indicate the total average of weights within and between the three intrinsic networks (DMN, CEN, SN), and, between the three networks and two subcortical regions (NAcc and Amyg) were obtained for each frame. The connectivity weight of the dynamic FC defined per pair of different regions for each frame per task condition was considered as the primary measure. The dynamic FC weight is denoted as *w*(*i*, *j*, *t*_1_, *t*_2_) in Figure 2, which conceptually illustrates how the measures were evaluated across the scan. The primary measure was weighted by HRF‐convolved time series that correspond to each task condition for evaluating task‐modulated effects in dynamic FC.

#### 2.8.2 Secondary DCC measures: Topological indexes

The topological properties of dynamic FC were considered as the secondary measures as calculated by Brain Connectivity Toolbox (Rubinov & Sporns, 2010). To quantify any topological properties, FC weights per timeframe were thresholded by setting originally negative values to zero, therefore resulting in only having non‐negative connectivity weights. In the topological indexes of connectivity, brain regions were referred to as *nodes*, and functional link between two of them as *edges*. Values of interest were network global efficiency (*G*_Eff_), deterministic modularity (*G*_Mod_) and topological resilience (*G*_Res_) that quantifies the reduction of network efficiency caused by removing high‐importance edges. Conceptually, global efficiency represents the degree of functional integration of the organization, and modularity that of segregation. Network resilience is in the trade‐off relationship with the efficiency of the network (Brede & de Vries, 2009; Netotea & Pongor, 2006).

Global efficiency of a network is a representative measure of how the information transfer is relatively easier across two topological locations (Latora & Marchiori, 2001). This metric is calculated as the summed reciprocals of the topological distance a node requires to connect itself to the other nodes within the network (Rubinov & Sporns, 2010), and a higher value indicates more efficient information transfer.

Deterministic modularity of a network is a representative measure of community structure of the network topology, which describes the density of connections between nodes inside each clusters that are sparsely linked to each other (Newman, 2006). This metric is calculated by quantifying the proportion of edges that connect nodes within each topologically divided clusters over those connect between the other clusters (Rubinov & Sporns, 2010). A higher value indicates more modular topology, or an organization that shows distinctly separated communities of nodes (Newman, 2006; Reichardt & Bornholdt, 2006).

Topological resilience is defined as a proxy measure of how much functional degradation a network structure can handle as described by Alstott, Breakspear, Hagmann, Cammoun, and Sporns (2009). The degradation in a network is conceptually modeled as the removal of an edge or edges, by setting the connectivity weight of the targeted edges and their transposed locations in the connectivity matrix to zero. Referring to Netotea and Pongor (2006), this paper quantified resilience (*G*_Res_) as the magnitude of change in global efficiency (*E*_100%_ – *E*_0%_) over the number of edges removed (*N*_100%_), multiplied by the total number of edges in the network (*N*_Total_ = 347 × 346/2 = 60,031 edges). In Equation (1), the 100% refers to the state of the connectivity matrix after removing all the targeted edges, and 0% the original state. The resilience is always negative, and higher values indicate more resilient structures. *N*_0%_ is equal to zero, thus not shown in the formula.

(1) $$G_{\mathrm{Res}}=\frac{E_{100\%}-E_{0\%}}{N_{100\%}}\times N_{\text{Total}}$$

According to Crossley et al. (2014), damage afflicted by many known brain disorders seem to center around the topologically most important nodes in the brain network. The number of shortest paths between any pair of nodes in the network that pass through a given edge is represented by a measure called edge betweenness centrality, and this measure represents that the edge is topologically more important for connecting nodes across the network efficiently (Freeman, 1978). Thus, edges with higher betweenness centrality are, by definition, likely to connect a functional hub with the other nodes. In this study, we simulated functional degradations in the brain network by removing all edges (100%) with the betweenness centrality values higher than one, leaving only the edges that have betweenness centrality values of either 0 (no shortest paths pass) or 1 (only one shortest path passes) in the original structure. Each topological index was weighted by HRF‐convolved task time series. The calculation of secondary measures was done on 120 high‐performance computing SLURM nodes with MATLAB R2018b.

### 2.9 Task‐modulated standard deviation of dynamic functional connectivity

Primary and secondary measures were quantified based on the connectivity matrix weighted by HRF‐convolved time series of task conditions (C1, W1, C2, W2, C3, and W3). The task‐modulated variability across time was the representative measure of this study, which was quantified by the *SD* (*d*_SD_) of dynamic FC. Equation (2) shows how *d*_SD_ of primary measures were calculated based on dynamic FC weighted by time series of task (*H*_task_), defined as the blocks of task convolved with canonical HRF *not* centered to zero mean.

(2) $$d_{\mathrm{SD}}=\frac{1}{T_{2}}\sum_{t_{2}=1}^{T_{2}}\left[\frac{1}{N_{\text{Conn}}}\sum_{j=1}^{J}\sum_{i=1}^{I}\sqrt{\left\{\frac{\sum_{t_{1}=1}^{T_{1}}{\left(w\left(i,j,t_{1},t_{2}\right)*H_{\text{task}}\left(t_{1},t_{2}\right)-E\left(d\right)\right)}^{2}}{T_{1}-1}\right\}}\right]$$

For the time series of the task and connectivity, *t*_1_ = {1 … *T*_1_} defines the temporal length in timeframes of one task trial (one of C1, W1, C2, W2, C3, or W3), and *T*_1_ is defined by the number of nonzero positive values within one trial window from *H*_task_; *t*_2_ = {1 … *T*_2_} represents the number of trials per task condition, which was set to 12 for all task conditions. The dynamic connectivity between region *i* and *j* at a fixed time point is denoted as *w*(*i*, *j*, *t*_1_, *t*_2_). Calculations in inner bracket performs the weighting (multiplication) of each dynamic connection values in accordance to *H*_task_, so that only the values that are relevant to task‐modulated BOLD activity are considered. The weighted connectivity is averaged across the number of connection pairs exist per type of connectivity (*N*_Conn_). *E*(*d*) represents the mean of weighted dynamic connectivity values of one pair across timeframes within one trial.

Task‐modulated *SD* aims to explore whether the nonstationary nature of the dynamic connectivity provides behavioral correlates of the task. The major difference of using dynamic FC compared to the generalized PPI (McLaren et al., 2012) is that it estimates the connectivity per pair of regions and per timeframe regarding the nonstationarity of the entire time series first (Lindquist et al., 2014), then calculates the task‐modulated connectivity weighted by task series, therefore eliminating the need of introducing a linear model of task‐based modulation that includes psychophysiological interactions. It is thus able to quantify the temporal variability of FC, which requires accounting for multiple timeframes within one trial of a task condition. Task‐modulated *SD* exploits the dynamic FC defined per timeframe to see if the task‐modulated magnitude of connectivity fluctuation can provide information on task‐modulated behavioral markers.

### 2.10 Statistical analyses and visualization

The statistical tests used in this study were independent *t*‐tests, repeated‐measures ANOVA, evaluation of sphericity in dependent variables using Mauchly's test, and nonparametric partial correlation (Spearman's rho, denoted as *R* for all figures). For repeated measures ANOVA, the statistical significance was determined from the results of multivariate analyses, then the main effect of task conditions within subjects for each dependent variable (FC measures) was evaluated using Greenhouse–Geisser's method with Bonferroni correction. The order of magnitude for each dependent variable across task conditions was also calculated using post hoc test with Bonferroni correction, and the order was represented as letter legends on figures. The main effect of group between subjects was further tested with post hoc Bonferroni correction for 12 connection weights for primary, and three topological indexes for secondary measures. Alpha level was defined at corrected *p* < .050 for all analyses. All the tests were performed using IBM SPSS (IBM Corp. Released 2016. IBM SPSS Statistics for Windows, Version 24.0. Armonk, NY). The three‐dimensional illustrations of brain changes were created using BrainNet viewer (Xia, Wang, & He, 2013). All the plots representing statistical results were created using R (R Core Team, 2013), with toolboxes of ggplot2 (Wickham, 2016), gridextra (Auguie, Antonov, & Auguie, 2017), and reshape2 (Wickham, 2012).

Statistical results for primary and secondary measures were presented as follows. For the primary measure, the *d*_SD_ regarding intrinsic networks and subcortical regions of Amyg and NAcc were compared between CON and CAN groups for six task conditions (C1, W1, C2, W2, C3, and W3) within each subject. The effects of interest were the main effect of task (within‐subject), group (between‐subject), and the interaction of task and group (within‐between). In the repeated measures ANOVA, dependent variables were dynamic primary measures of 12 connectivity types. Thus, multiple comparison corrections were applied using Bonferroni's method for 12 cases. For the secondary measure, the *SD* of topological properties (*G*_Eff_, *G*_Mod_, and *G*_Res_) were analyzed as dependent variables affected by the same effects of interest. Multiple comparison corrections were applied using Bonferroni's method for three cases. Both models included age and the average framewise displacement across a total 810 dynamic timeframes (mean‐centered per group, CON and CAN) as nuisance covariates to account for the error variance. The main effect of age is known to significantly alter connectivity‐related measures (Ferreira et al., 2016), and considering average framewise displacement may reduce the potential effect of head motion upon the connectivity measures (Siegel et al., 2016).

A post hoc correlation analysis between each dynamic connectivity measures and subjective craving within CAN was performed. Nonparametric partial correlation using Spearman's rho was performed for primary and secondary measures, correcting for the variance of age and average framewise displacement. Multiple comparison corrections were performed using false discovery rate (FDR) of 25% under a restriction that the raw *p* values are smaller than .050 (Benjamini & Hochberg, 1995).

## 3 RESULTS

### 3.1 Group and task condition effects on dynamic functional connectivity

#### 3.1.1 Primary measures: Functional connectivity weights

**FIGURE 3:**
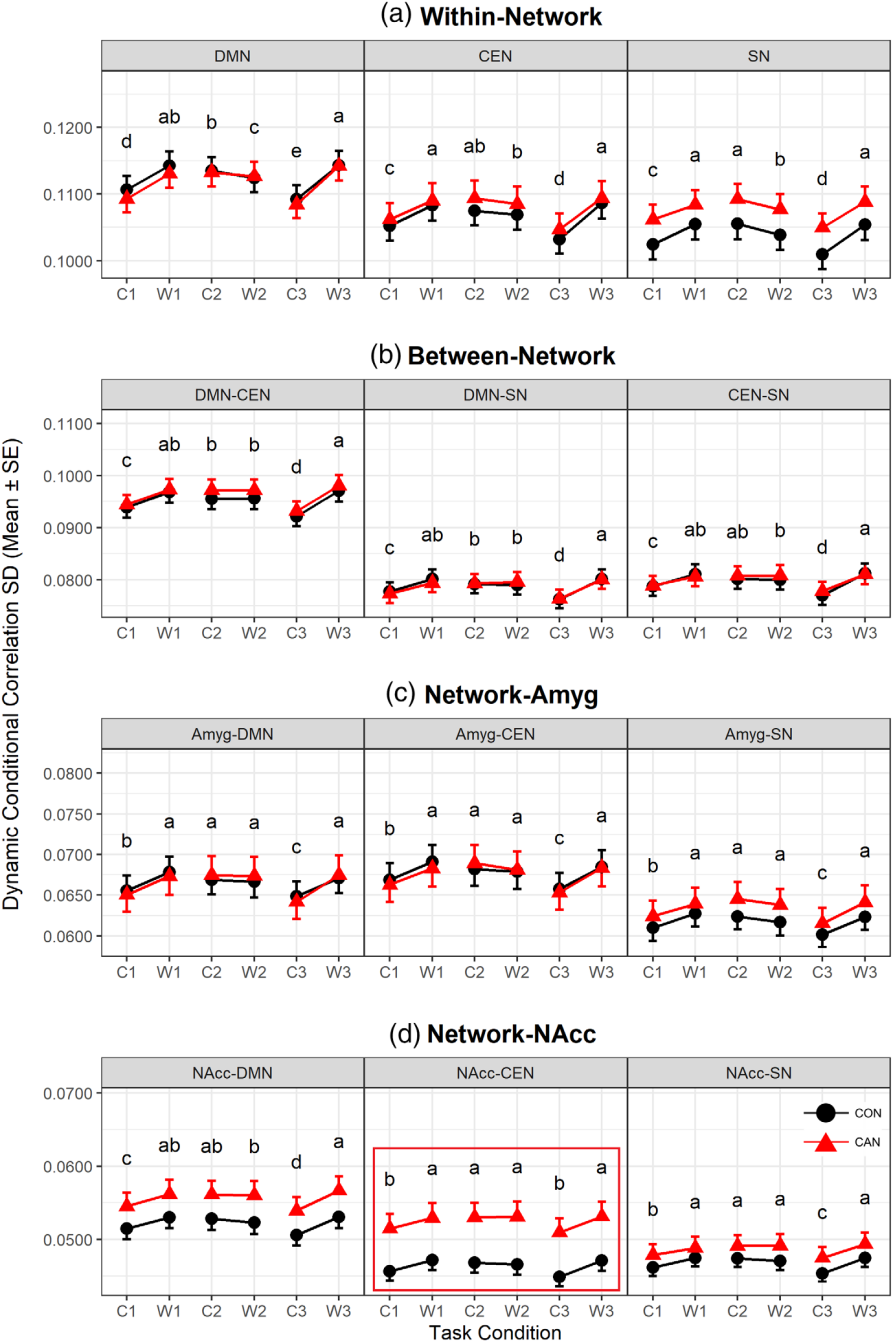
The standard deviation of task‐modulated primary measure of dynamic functional connectivity in the healthy control and cannabis users (CON vs. CAN). Markers indicate standard deviation of the primary measures per group, and error bars denote the standard error (*n* = 90 for CON, *n* = 54 for CAN). The *X*‐axis represents task conditions (C1, W1, C2, W2, C3, and W3) and *Y*‐axis the magnitude of the present measure. The *Y*‐axis is shared for the same row of three plots. A subplot that showed significant group effect is highlighted with red boundary. Abbreviations are default mode network (DMN), central executive network (CEN), salience network (SN), amygdalae (Amyg), and nuclei accumbens (NAcc). Task conditions abbreviated are neutral cue ON (C1), neutral cue OFF (W1), natural reward cue ON (C2), natural reward cue OFF (W2), cannabis cue ON (C3), and cannabis cue OFF (W3). Black circles indicate healthy controls (CON), and red triangles cannabis users (CAN)

There was a significant effect of task on *d*_SD_, showing *F*(60, 3,465) = 14.979, *p* < .001. After multiple comparison correction, task‐modulated differences were found to be significant for all connectivity types of interest. The group effect was significant on changes in *d*_SD_, with *F*(12, 129) = 2.050, *p* = .025. After Bonferroni correction for individual between‐subject effects, the *d*_SD_ of NAcc‐CEN was found to be higher in CAN when averaged across task conditions, showing *F*(1, 140) = 8.502, uncorrected *p* = .0041, corrected *p* = .0496. The interaction of group and task effects was not significant, showing *F*(60, 3,465) = 1.204, *p* = .136. Figure 3 represents the task‐modulated *d*_SD_ differences of primary measures by task conditions and groups after Bonferroni correction applied.

#### 3.1.2 Secondary measures: Topological indexes

**FIGURE 4:**
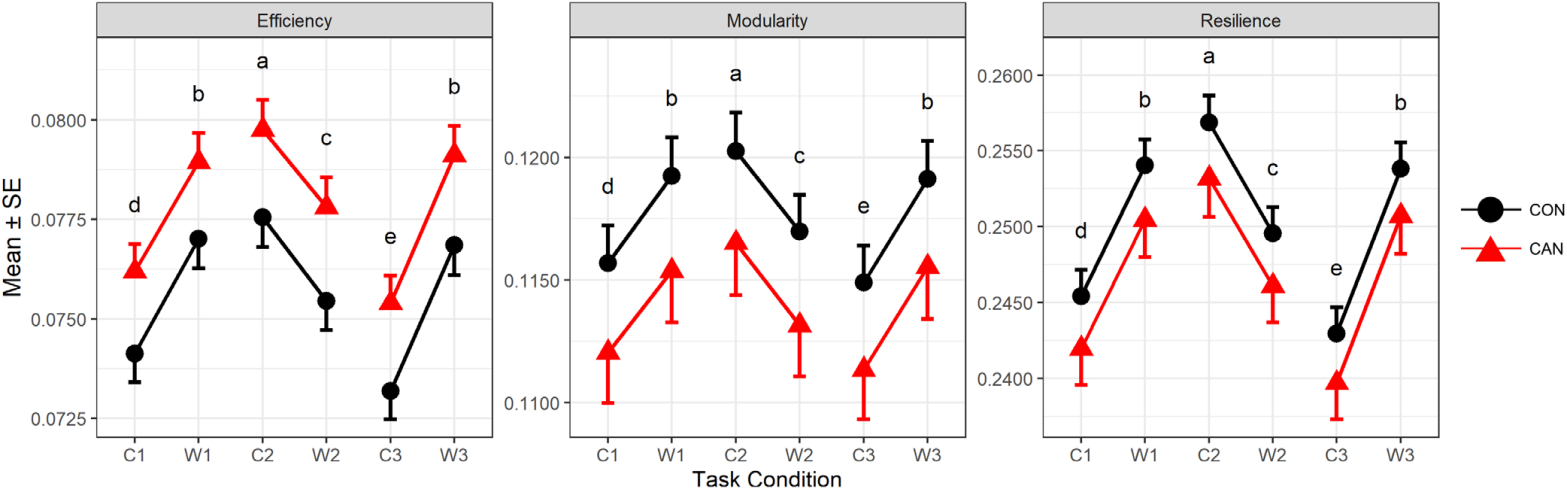
The standard deviation of task‐modulated secondary measure of dynamic functional connectivity in the healthy control and cannabis users (CON vs. CAN). Markers indicate standard deviation of the secondary measures per group, and error bars denote the standard error (*n* = 90 for CON, *n* = 54 for CAN). The *X*‐axis represents task conditions (C1, W1, C2, W2, C3, and W3) and *Y*‐axis the magnitude of the present measure. The *Y*‐axis is not shared across the plots. Black circles indicate healthy controls (CON), and red triangles cannabis users (CAN)

The effect of task on secondary measures’ *d*_SD_ was significant (*F*[15, 2,100] = 68.837, *p* < .001). The group effect was significant (*F*[3, 138] = 2.906, *p* = .037), but individual effects did not survive multiple correction (maximum *F*[1,140] = 4.584, minimum uncorrected *p* = .034, corrected *p* = .102 for *G*_Eff_). The interaction was not significant (*F*[15, 2,100] = 1.009, *p* = .442). Figure 4 represents task‐modulated *d*_SD_ differences of secondary measures by task conditions and groups after Bonferroni correction applied.

### 3.2 Correlation of subjective craving with dynamic functional connectivity

#### 3.2.1 Subjective craving with primary measures

**FIGURE 5:**
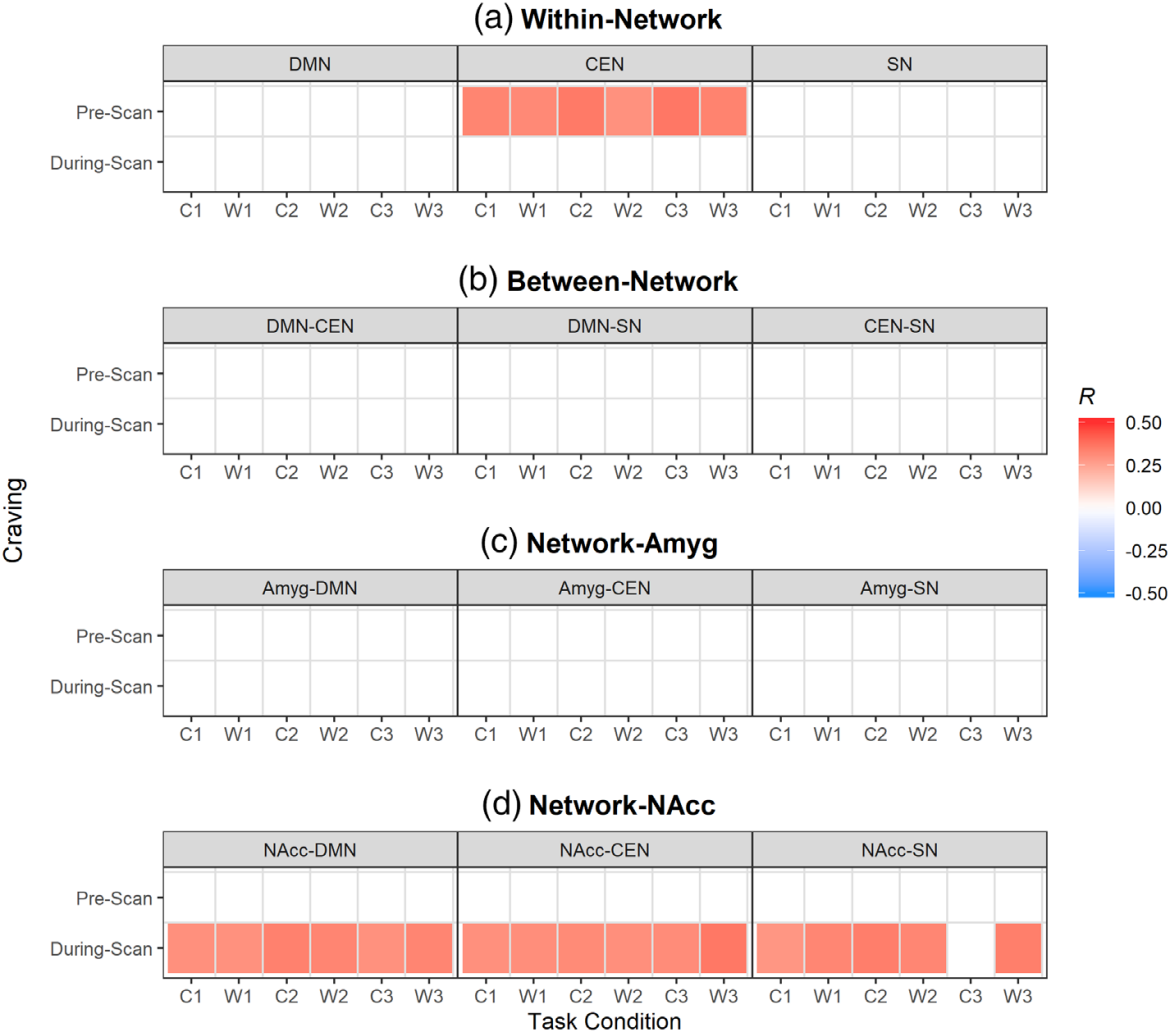
Correlation of standard deviation of primary measure in dynamic functional connectivity with craving scores in cannabis users (CAN). Correlation coefficients that survive the multiple comparison correction using FDR *q* ≤ 0.250 and uncorrected *p* < .050 (out of 144 cases) are shown as colored boxes. Each box is color‐coded to represent the direction of correlation (Spearman's rho), where red is positive and blue is negative. The color scale is identical across all types of primary measures. The *X*‐axis represents task conditions (C1, W1, C2, W2, C3, and W3) and *Y*‐axis the craving scores in the order of prescan (subject *n* = 52) and during‐scan (*n* = 54). Abbreviations indicate default mode network (DMN), central executive network (CEN), salience network (SN), amygdalae (Amyg), and nuclei accumbens (NAcc). Task conditions abbreviated are neutral cue ON (C1), neutral cue OFF (W1), natural reward cue ON (C2), natural reward cue OFF (W2), cannabis cue ON (C3), cannabis cue OFF (W3)

The *d*_SD_ of NAcc‐DMN and NAcc‐CEN connectivity for all conditions was significantly correlated with during‐scan craving in the positive direction. The *d*_SD_ of NAcc‐SN connectivity for all conditions was significantly correlated with during‐scan craving in the positive direction as well, but C3 condition did not pass the multiple correction (uncorrected *p* < .05, *q* > 0.25). The *d*_SD_ of WithinCEN connectivity for all conditions showed a positive correlation with the prescan craving. Figure 5 represents the direction and the magnitude of correlation coefficients for *d*_SD_ of primary measures and craving scores. The worst case that passes FDR correction was NAcc‐SN with during‐scan craving in C1 condition (uncorrected *p* = .038, *q* = 0.238).

#### 3.2.2 Subjective craving with secondary measures

**FIGURE 6:**
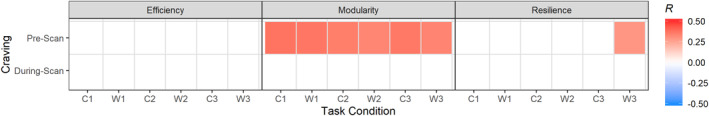
Correlation of standard deviation of secondary measure in dynamic functional connectivity with craving scores in cannabis users (CAN). Correlation coefficients that survive the multiple comparison correction using FDR *q* ≤ 0.250 and uncorrected *p* < .050 (out of 36 cases) are shown as colored boxes. Each box is color‐coded to represent the direction of correlation (Spearman's rho), where red is positive and blue is negative. The color scale is identical across all types of secondary measures. The *X*‐axis represents task conditions (C1, W1, C2, W2, C3, and W3) and *Y*‐axis the craving scores in the order of prescan (subject *n* = 52) and during‐scan (*n* = 54)

The *d*_SD_ of *G*_Mod_ was correlated with the prescan craving in positive direction for all conditions. Figure 6 accounts for the direction and the magnitude of correlation coefficients for *d*_SD_ of secondary measures and craving scores. The worst case that passes FDR correction was *G*_Res_ in W3 condition with prescan craving (uncorrected *p* = .043, *q* = 0.221).

## 4 DISCUSSION

In this study, we examined the effects of task and cannabis use on the dynamic FC of the brain during a cue exposure task. Our findings showed that (a) the variability of NAcc‐CEN connectivity weights was significantly higher in cannabis users than controls regardless of task conditions, (b) the variability within the CEN was positively correlated with prescan craving, (c) the variability of dynamic FC between NAcc and the two major intrinsic networks, the DMN and the CEN, was positively correlated with during‐scan craving, and (d) the variability of whole‐brain network modularity was positively correlated with prescan craving. Both pre‐ and during‐scan subjective craving ratings were associated with the system‐level changes in the brain in a temporally dynamic manner that is not evaluated through static connectivity analyses. This study distinguished dynamic FC differences involving reward‐related regions associated with cannabis use and linked the subjective craving in cannabis users with changes in the variability of dynamic connectivity measures. The results complement previous knowledge on static FC, and potentially add systematic evidences to the known models of SUDs from a specific case of cannabis use.

### 4.1 Temporally dynamic nature of task‐modulated connectivity during cue‐reactivity

The concept of dynamic FC represents the nonstationary nature of connectivity, the mean and variance of which is altered by the internal functional state of the brain (Chang & Glover, 2010), such as the level of consciousness (Barttfeld et al., 2015), alertness (Chang, Liu, Chen, Liu, & Duyn, 2013), sleep stages (Tagliazucchi & Laufs, 2014), or task conditions (Braun et al., 2015; Fong et al., 2019). Drug cue exposure is likely to affect the BOLD signal in the DA reward pathway, or mesocorticolimbic pathway (Filbey et al., 2009, 2016; Filbey & Dunlop, 2014), which may lead to BOLD‐derived FC changes. The mesocorticolimbic pathway originates from the midbrain (ventral tegmental area and substantia nigra) that elicits DA modulation upon NAcc (Yun, Wakabayashi, Fields, & Nicola, 2004) and the prefrontal cortex (Lewis & O'Donnell, 2000). We therefore expected that the dynamic nature of cue‐induced DA signal changes in the midbrain underlies the changes in dynamic FC.

Upon the exposure to cannabis cues for cannabis users, DA signals in the mesocorticolimbic reward pathway may increase (Berridge & Robinson, 2016), potentially reflecting the increased salience attribution and motivated processing of information in the users (Littel, Euser, Munafo, & Franken, 2012; Norberg, Kavanagh, Olivier, & Lyras, 2016). In this study, we found that the main effect of task is significant for the variability of both primary and secondary measures. Further, it clearly delineated across different task conditions during the cue exposure task, suggesting that the nonstationarity of FC changes is highly relevant to the main effect of the task upon FC measures. This may imply that the dynamic changes in DA signals are transient so that they may not be fully estimated using the static connectivity model.

There are tonic and phasic activity patterns of DA neurons; while tonic activity represents a steady frequency of firing, phasic shows a transiently faster, burst‐then‐pause rhythm of firing (Floresco, West, Ash, Moore, & Grace, 2003). Phasic activity induces a surging increase of DA release (Gonon, 1988), and is known to facilitate reward‐motivated reinforcement learning (Goto & Grace, 2005; Grace, Floresco, Goto, & Lodge, 2007; Wolfram Schultz, 1997). An optogenetics study has shown that phasic activity is sufficient for behavioral conditioning (Tsai et al., 2009), which is a key process related to SUDs (Everitt, Dickinson, & Robbins, 2001). Phasic DA activity in NAcc is known to increase in a temporally associated manner in rats trained to self‐administer cocaine toward drug‐related cues, along with the actual increase of DA release in the NAcc core (Ito, Dalley, Howes, Robbins, & Everitt, 2000; Stuber, Roitman, Phillips, Carelli, & Wightman, 2005). On the contrary, the same activity seems to decrease during withdrawal period in rats chronically exposed to THC (Diana, Melis, Muntoni, & Gessa, 1998). This implies that the modulation of reward‐related DA signals in NAcc of cannabis users fluctuates. It further corroborates that the DA signal changes in cue‐reactivity will be more dynamic for cannabis users compared to controls, which may have led to increased variability of dynamic connectivity in users. We did not find a group × task interaction effect, which suggests that task‐modulated changes in DA signals did not affect the dynamic FC to an extent that can overcome the group differences.

### 4.2 Variability in functional connectivity weights

The variability of dynamic FC weights between NAcc and the CEN was found to be higher in the CAN group. NAcc and the CEN have generalizable and important implications in SUDs, especially in addressing behavioral aspects via the addiction model of Impaired Response Inhibition and Salience Attribution (iRISA; R. Z. Goldstein & Volkow, 2011; Zilverstand et al., 2018). This model aims to explain the mechanisms of SUDs that correspond to behavioral stages of binge‐intoxication, withdrawal‐negative affect, and preoccupation‐anticipation (Koob & Volkow, 2016). The functional underpinning of this cycle is hypothesized to be the downregulated executive control that modulates response inhibition (R. Z. Goldstein & Volkow, 2011) and imbalanced salience attribution that focuses on drug‐related cues above others (Droutman, Read, & Bechara, 2015). Our finding showed that the temporal link between subcortical reward locus (NAcc) and the CEN involved in response inhibition fluctuates more in the cannabis users, supporting the iRISA model by showing the neural marker of significant interplay between two functions supposedly imbalanced. This systematic evidence based on cannabis users might be applicable to the other types of SUDs that share the behavioral aspects of the development.

The loss of executive or inhibitory control that leads to impulsive action is prominent in SUDs (Bari & Robbins, 2013), and it causes susceptibility toward the onset and further use of illicit drugs (Brockett, Pribut, Vázquez, & Roesch, 2018; Dalley, Everitt, & Robbins, 2011; Dalley & Robbins, 2017). The CEN and the SN are two major intrinsic networks involved in this function, potentially reorienting attention based on task objectives or the salience of information, respectively (Corbetta, Patel, & Shulman, 2008; Dalley & Robbins, 2017; Dodds, Morein‐Zamir, & Robbins, 2010). These two networks seem to collaborate in response to environmental stressors in general, including reward‐related situations that subsequently induce DA impulse, to react and process the information (Hermans, Henckens, Joels, & Fernandez, 2014). The CEN is more involved in goal‐oriented, or task‐modulated processing that aids decision making (Dodds et al., 2010; Menon, 2011) and, importantly, response inhibition (Chikazoe et al., 2009; Dodds et al., 2010; van Gaal, Ridderinkhof, Scholte, & Lamme, 2010). Functions of NAcc support the subjective appraisal of value by putting incentive into consideration (Milton & Everitt, 2012), and its neural activity relates to salience and reward (Cooper & Knutson, 2008; Horvitz, 2000).

Neural responses of NAcc and the CEN both appear to imply changes in craving or reward‐related decision making. For substance‐dependent users, the neural activity in NAcc increases in response to drug‐related cues (Koob & Volkow, 2010), and cannabis users with more problematic symptoms show a higher activity here (Cousijn et al., 2013). The activity in NAcc seems to decrease in relation to downregulated craving toward cigarette smoking (Kober et al., 2010). On the other hand, the higher activity in regions in the CEN was correlated with lower craving in cigarette smokers (Kober et al., 2010) and cannabis users (Cousijn et al., 2013). Furthermore, FC within the CEN may decrease in the resting state for chronic stimulant users, while it may increase within the mesocorticolimbic reward pathway (including NAcc), and between the CEN and the reward pathway (Zilverstand et al., 2018). In sum, the pattern of activity and connectivity changes in the reward pathway and the CEN may be the opposite at the same functional state. The variability of dynamic FC between two may thus reflect their more fluctuating interplay due to their distinct differences in the functional states.

The increased dynamic connectivity variability in cannabis users may have been affected by the fluctuations of DA signals between cue ON and OFF states. The change was found to be independent of task conditions, possibly because it reflects the dysfunctional modulation of DA signal, not a mere increase or a decrease in the magnitude (see Supplementary Results for findings in the average of dynamic connectivity). The DA signal in NAcc is known to be a dynamic modulator of motivated behaviors in general (Salamone, Correa, Mingote, & Weber, 2005; W. Schultz, 2007), and an impairment to NAcc core leads to impulsivity rather than the loss of motivated behaviors (Cardinal, Pennicott, Lakmali, Robbins, & Everitt, 2001), which relates to SUDs (Brockett et al., 2018; Dalley et al., 2011; Dalley & Robbins, 2017). Originally, the DA signal from the midbrain that projects to the lateral prefrontal cortex in the CEN aids the cognitive control of action not by weight information by the reward value, but rather by providing the salience signal (Ott & Nieder, 2019). However, while the reward pathway including NAcc is activated by immediately available rewards, regions in the CEN are activated by delayed rewards that require more cognitive resource to eventually make a less impulsive decision (McClure, Laibson, Loewenstein, & Cohen, 2004). These literatures imply that the executive control of the CEN over reward‐related information such as cannabis cues is hierarchically positioned higher and is activated depending on the current state of “wanting” represented by the reward pathway. The striatum and the prefrontal cortex functionally interact for reward‐related decision making (Cools, 2015). DA signal seems to provide the goal‐oriented flexibility in NAcc (Haluk & Floresco, 2009), and the stability by concentrating on goals in prefrontal cortex (Bloemendaal et al., 2015; Noudoost & Moore, 2011). Chronic cannabis use can induce dysfunctional modulation of DA signals and FC of NAcc (Manza, Tomasi, & Volkow, 2018; van de Giessen et al., 2017; Volkow et al., 2014) and aberrances in activity and connectivity within the CEN (Krmpotich et al., 2013; Tapert et al., 2007). It can further weaken the interaction between the prefrontal cortex and NAcc (Fischer, Whitfield‐Gabrieli, Roth, Brunette, & Green, 2014; Hwang & Lupica, 2019). In sum, the increased variability between NAcc and the CEN in cannabis users may indicate the lack of stable modulation upon the reward‐related DA signal.

We found a positive correlation of dynamic connectivity variability within the CEN with the prescan (baseline) craving. This correlation may imply the involvement of the dysfunctional CEN in managing the craving (Luijten et al., 2014; Zilverstand et al., 2018), reflecting the higher demand of cognitive control over the transient fluctuations of DA signals. Dynamic connectivity variability from NAcc to the three intrinsic networks showed significant positive correlation with the during‐scan craving regardless of the task conditions, except in NAcc‐SN where cannabis cue ON condition did not pass the multiple correction. This probably indicates that the fluctuations of DA signals in NAcc are constantly affecting some of the major large‐scale intrinsic networks. The during‐scan craving may relate to the relapse of substance use (Crombag, Bossert, Koya, & Shaham, 2008; Grimm, Hope, Wise, & Shaham, 2001). Two of the neural underpinnings of the discrepancy between prescan and during‐scan cravings are the loss of DA signals during withdrawal (Diana et al., 1998) and the cue‐induced phasic DA signals (Berridge & Robinson, 2016), both of which can increase the fluctuation of the DA signal over time. This suggests that the during‐scan craving may be more dependent on the dysfunctional modulation of striatal DA signals. Meanwhile, the variability of dynamic FC between NAcc and the three intrinsic networks was not correlated with the prescan craving, which may also imply that the effect of unstable modulation of DA signals is subject to external treatment to reduce craving during the cue exposure task, but not the abstinence period. Perhaps targeting the CEN may be more effective in managing the craving during cannabis abstinence, because of the relevance of the variability of dynamic FC to prescan craving.

### 4.3 Variability in topological indexes

The most prominent finding is that the pattern of changes between states of natural reward cue ON versus OFF is the opposite of what is observed in neutral and cannabis cue states. This contrast is clearer than that from the dynamic primary measures in this study. The substance‐dependent users show markers of higher allocation of attention to substance cues than neutral cues regardless of substance types and whether abstinence was on (Littel et al., 2012). The extant addiction models hypothesize that the positive reinforcement caused by drugs of abuse acts the same as that by natural rewards, and overrides the reward effect from nondrug gains by abnormally highlighting the reward of drug (Kelley & Berridge, 2002). In a cue exposure task, natural reward cues are introduced to distinguish the inherent motivational relevance from what is caused by the effect of substances (Versace et al., 2017). The current study showed that the pattern of task‐modulated variability of topological reorganization during exposure to cannabis cues appears to be significantly different from that found with natural reward cues, but like that with neutral cues. The lack of group‐task interaction suggests that this effect is not unique for the cannabis users. The present result represents the systematic marker of dynamic FC that highlights the differences in the neural underpinning for processing natural reward compared to the conditioned reward (cannabis) or neutral information, regardless of chronic cannabis use.

Previous studies have suggested that dynamic FC appear to denote an adaptive change in interaction within and between different functional modalities based on given tasks (Braun et al., 2015). This includes large‐scale changes, such as; linking more to the prefrontal network, that is, the CEN, for tasks that require executive controls (Braun et al., 2015; Cole, Reynolds, et al., 2013); connecting regions in the DMN across the brain for more global integration of information (Vatansever, Manktelow, Sahakian, Menon, & Stamatakis, 2017; Vatansever, Menon, Manktelow, Sahakian, & Stamatakis, 2015) and; in terms of topological features of the FC, adjusting the roles of the most densely connected brain hubs to increase the network efficiency at the moment (de Pasquale et al., 2016). Topological reorganization of the connectivity seems to alter the network efficiency (integration) and modularity (segregation) depending on the types of task demands (Cohen & D'Esposito, 2016). Liégeois et al. (2019) used Human Connectome Project datasets to show that dynamic connectivity provides more information for explaining variances of task‐modulated behaviors. During tasks, DA is known to actively and dynamically modulate the sensory information and the executive functions in the prefrontal cortex (Ott & Nieder, 2019). Taken together, the dynamic topological reorganization in the brain may be affected by the task‐modulated activity of DA, probably in the prefrontal cortex. The differential pattern of changes in dynamic FC variability found during exposure to natural reward cues could indicate that the roles of DA signal are also distinct compared to when exposed to the other cues. A further study is needed to clarify the neural underpinning of this phenomenon, especially testing for the effect of abstinence, and the type of illicit drugs of abuse.

We found that as the variability of network modularity increase, so does prescan craving. Network modularity conceptually represents the degree of modal and segregated communications across brain regions (Cohen & D'Esposito, 2016). Higher modularity is prominent during simpler motor tasks (Cohen & D'Esposito, 2016), and relates to higher perceptual acuity in a sensory task (Sadaghiani, Poline, Kleinschmidt, & D'Esposito, 2015). Lower modularity, on the other hand, reflects less segregated network organization, and seems to be a marker for the functional state favorable for the awareness of the target information (Godwin, Barry, & Marois, 2015). A fluctuating modularity across conditions therefore indicates an unstable state switching between functional modes apt for distinct tasks. Since our task has not given goals that require cognitive load, this fluctuation cannot be due to internal adaptation for the task performance. It appears that after the external administration of L‐DOPA, network modularity decreases (Alavash et al., 2018) and the connectivity between subcortical regions and cortical networks may increase (D. M. Cole, Oei, et al., 2013), leading the functional structure to be more integrated than segregated. This may imply that during the cue exposure task under higher prescan craving, the DA modulation upon the brain network is less stable in cannabis users. The current results suggest that the unstable segregation of the brain network relates to the loss of DA signal balance, which is potentially more disrupted in cannabis users than the controls.

### 4.4 Limitations and suggestions for future studies

One of this study's limitations is that the subjective level of craving can be affected by several factors not fully quantified in this study. Two of the major examples are hormonal changes associated with menstrual period in female, and the time of day. Sherman, Caruso, and McRae‐Clark (2019) found that an external administration of progesterone may attenuate abstinence‐induced craving for cannabis (marginally significant effect), suggesting that the level of female hormone can affect the level of subjective craving. In addition, when the craving is present, time of day had a significant main effect upon its subjective level (Shrier, Walls, Kendall, & Blood, 2012). These can be controlled for identifying a clearer effect of craving in future studies. Second, it is another limitation that the potential covariate effect of the comorbidity of nicotine use and the cannabis use, was not explored in this study. A future investigation with a larger sample size of the cannabis users, and a control group that includes a sizable number of nicotine users may be able to substantiate the effect.

Lindquist et al. (2014) introduced DCC and noted its lower precision for handling short‐term state changes in the fMRI signal. Although this study used a block design in the cue‐exposure task (cue exposure and washout for 20 s), whose effect may account for longer‐term changes in functional state, they may still be underestimated according to Lindquist et al. (2014). We considered the transient effect in dynamic FC by weighting the resultant primary and secondary measures with HRF‐convolved time series to avoid a brief‐term effect at the very beginning and the end of each block so that changes DCC was slow to detect minimally bias our results. The present results not only show significant task effect across conditions, but also a consistent increase in the *SD* of DCC in CAN group for NAcc‐CEN regardless of task conditions. Thus, our main results are less likely to have been compromised by the underestimation for brief changes. Nevertheless, it is certainly possible that rapid states including emotional disturbance or arousal in attention would be present in cannabis users upon cue exposure. To understand these functional effect, more elaborate task design should be accompanied with an alternative metric for dynamic FC, that is, dynamic connectivity regression (Cribben, Haraldsdottir, Atlas, Wager, & Lindquist, 2012), which Lindquist et al. (2014) has referred to.

The association of cognitive functions with the cannabis use, especially focusing on the functions of response inhibition and salience attribution, may be further studied. The study will support the link of dynamic FC alterations with the central functional effect of substance use in accordance to the iRISA model. In the same context, the task‐modulated role of the DMN, which relates to the processing of internal information, or the nontask states (Raichle, 2015), may be further investigated to verify its roles separate from the CEN in more cognitive tasks. Recently, Zhang and Volkow (2019) extensively reviewed evidences showing that the impairment of DA signaling can prevent the normal functions of the DMN, and the interplay of the DMN with the CEN and the SN are disrupted in SUDs. Dynamic FC studies that integrate the general implications of the DMN to the addiction models are desired.

## 5 CONCLUSIONS

Utilizing the dynamic functional connectivity, we were able to quantify the task‐modulated variability of connectivity weights and topological indexes over time. The current results identified the system‐to‐behavior level link in cannabis use based on the correlational analysis of dynamic functional connectivity and subjective craving. Our findings suggest that the alterations in the variability of dynamic connectivity in cannabis users reflect their underlying dysfunctions in modulating dopamine system in molecular level that is constantly present in long‐term users, and that the dysfunctions affect large‐scale intrinsic networks via subcortical to cortical link.

## CONFLICT OF INTEREST

The authors report no conflict of interest.

## Supporting information


**Data S1**: Supporting information

**Figure S1** *Visualization of the intrinsic networks of interest*. Each spheres indicate the geometric centroids of the brain regions mapped on the cortex, as defined in Gordon et al. (2016). Three networks are delineated by black (default mode network), blue (central executive network), and red (salience network) colors.

**Figure S2** *Craving for cannabis use upon exposure to neutral*, *natural reward*, *and cannabis cues*. Lines represent the raw mean score of craving (minimum 0 to maximum 10) for all cannabis users (CAN, *n* = 54), across 12 trials from two sessions. The shaded area denotes 95% confidence interval across participants.

**Figure S3** *The mean of task‐modulated primary measure of dynamic functional connectivity in the healthy control and cannabis users (CON* vs. *CAN)*. Markers indicate mean of the primary measures per group, and error bars denote the standard error (*n* = 90 for CON, *n* = 54 for CAN). The *X*‐axis represents task conditions (C1, W1, C2, W2, C3, and W3) and *Y*‐axis the magnitude of the present measure. The *Y*‐axis is shared for the same row of three plots. Abbreviations indicate default mode network (DMN), central executive network (CEN), salience network (SN), amygdalae (Amyg), and nuclei accumbens (NAcc). Task conditions abbreviated are neutral cue ON (C1), neutral cue OFF (W1), natural reward cue ON (C2), natural reward cue OFF (W2), cannabis cue ON (C3), cannabis cue OFF (W3). Black circles indicate healthy controls (CON), and red triangles cannabis users (CAN).

**Figure S4** *The mean of task‐modulated secondary measure of dynamic functional connectivity in the healthy control and cannabis users (CON* vs. *CAN)*. Markers indicate mean of the secondary measures per group, and error bars denote the standard error (*n* = 90 for CON, *n* = 54 for CAN). The *X*‐axis represents task conditions (C1, W1, C2, W2, C3, and W3) and *Y*‐axis the magnitude of the present measure. The *Y*‐axis is not shared across the plots. Black circles indicate healthy controls (CON), and red triangles cannabis users (CAN).

**Figure S5** *Correlation of mean of primary measure in dynamic functional connectivity with craving scores in cannabis users (CAN)*. Correlation coefficients that survive the multiple comparison correction using FDR *q* ≤ 0.250 and uncorrected *p* < .050 (out of 144 cases) are shown as colored boxes. Each box is color‐coded to represent the direction of correlation (Spearman's rho), where red is positive and blue is negative. The color scale is identical across all types of primary measures. The *X*‐axis represents task conditions (C1, W1, C2, W2, C3, and W3) and *Y*‐axis the craving scores in the order of prescan (*n* = 52) and during‐scan (*n* = 54). Abbreviations indicate default mode network (DMN), central executive network (CEN), salience network (SN), amygdalae (Amyg), and nuclei accumbens (NAcc). Task conditions abbreviated are neutral cue ON (C1), neutral cue OFF (W1), natural reward cue ON (C2), natural reward cue OFF (W2), cannabis cue ON (C3), cannabis cue OFF (W3).

**FIGURE S6** Correlation of mean of secondary measure in dynamic functional connectivity with craving scores in cannabis users (CAN). Correlation coefficients that survive the multiple comparison correction using FDR *q* ≤ 0.250 and uncorrected *p* < .050 (out of 36 cases) are shown as colored boxes. Each box is color‐coded to represent the direction of correlation (Spearman's rho), where red is positive and blue is negative. The color scale is identical across all types of secondary measures. The *X*‐axis represents task conditions (C1, W1, C2, W2, C3, and W3) and *Y*‐axis the craving scores in the order of prescan (*n* = 52) and during‐scan (*n* = 54).

**Figure S7** *The standard deviation of task‐modulated primary measure of dynamic functional connectivity in the non‐ or little‐smoking subpopulation in healthy control and cannabis users (CON* vs. *CAN)*. Markers indicate mean of the primary measures per group, and error bars denote the standard error (*n* = 77 for CON, *n* = 27 for CAN). The *X*‐axis represents task conditions (C1, W1, C2, W2, C3, and W3) and *Y*‐axis the magnitude of the standard deviation between central executive network (CEN) and nuclei accumbens (NAcc). Task conditions abbreviated are neutral cue ON (C1), neutral cue OFF (W1), natural reward cue ON (C2), natural reward cue OFF (W2), cannabis cue ON (C3), cannabis cue OFF (W3). The red box indicates the significant main effect of group. Black circles indicate healthy controls (CON), and red triangles cannabis users (CAN).

## Data Availability

Data and codes used in this study are available from the corresponding author upon request.
