# Supplementary material for: Dynamic functional connectivity between nucleus accumbens and the central executive network relates to chronic cannabis use
Source: Hum Brain Mapp. 2020 May 20;41(13):3637–54. doi: 10.1002/hbm.25036 (PMC7416060; doi:10.1002/hbm.25036)
Supplement: Supplementary file 1 — Data S1: Supporting information [file HBM-41-3637-s002.docx]

# Supplementary Methods

## Consistency in Cue-Evoked Craving

A repeated measures ANOVA was applied to determine if during-scan cravings for cannabis upon the exposure to neutral, natural reward, and cannabis cues were constant across time. A general linear model was constructed using cue task trial and type as independent variables (IVs) and cue craving scores as dependent variables (DVs), and the statistical significance was evaluated using Greenhouse-Geisser’s method. The within-subject effects of interest were for the trials (12 trials total) and cue types (three types) within the CAN group (*n* = 54).

## Task-Modulated Mean of Dynamic Functional Connectivity

### Definition

The task-modulated average across time was additionally evaluated using the mean (*d_mean_*) of dynamic functional connectivity (FC) for all task conditions (C1, W1, C2, W2, C3 and W3). Eq. 1 shows how *d_mean_* of primary measures were calculated based on dynamic FC weighted by time series of task (*H_task_*), defined as the blocks of task convolved with canonical HRF *not* centered to zero mean.

| $d_{mean}= \frac{1}{T_{1}*T_{2}}\sum_{t_{2}=1}^{T_{2}} \sum_{t_{1}=1}^{T_{1}} \left[ \frac{1}{N_{Conn}}\sum_{j=1}^{J} \sum_{i=1}^{I} \left\{ w\left( {i,j,t}_{1},t_{2} \right)*H_{task}(t_{1},t_{2}) \right\} \right]$ | (Eq. 1) |
| --- | --- |

For the time series of the task and connectivity, *t_1_* = {1… *T_1_*} defines an index of the timeframes of one task trial (one of C1, W1, C2, W2, C3 or W3), and *T_1_* is defined by the number of non-zero positive values within one trial window from *H_task_*; *t_2_* = {1… *T_2_*} defines and index of the trial within one task condition, and *T_2_* is defined by the number of trials within one task condition, which was set to 12 for all task conditions. The dynamic connectivity between region *i* and *j* at a fixed time point is denoted as *w*(*i*, *j*, *t_1_*, *t_2_*). Calculations in inner bracket performs the weighting (multiplication) of each dynamic connection values in accordance to *H_task_*, so that only the values that are relevant to task-modulated BOLD activity are considered. The weighted connectivity is averaged across the number of connection pairs that exist per type of connectivity (*N_Conn_*). Task-modulated mean aims to reflect the non-stationary nature of the dynamic connectivity in terms of varying mean across time, and provides more direct reflection of the averaged momentary connectivity for a task condition without the need for introducing a linear model of task-based modulation, as in psychophysiological interactions ([Friston et al., 1997](#_ENREF_6); [McLaren, Ries, Xu, & Johnson, 2012](#_ENREF_11)).

### Statistical Analyses

The same methods as in the main manuscript were applied. Repeated measures ANOVA was used to identify the main effect of task (within-subject), group (between-subject), and the interaction of task and group (within-between). The statistical significance was first determined from the results of multivariate analyses using Pillai’s trace, then the main effect of task conditions within subjects for each dependent variable was evaluated using Greenhouse-Geisser’s method with Bonferroni correction, and the order of magnitude was calculated using a post-hoc test with Bonferroni correction. The main effect of group between subjects was further tested with post-hoc Bonferroni correction for 12 connection weights (FC regarding intrinsic networks – DMN, CEN, SN and subcortical regions of Amyg and NAcc) for primary measures, and for 3 topological indexes (*G_Eff_*, *G_Mod_* and *G_Res_*) for secondary measures. Both models included age and the average framewise displacement across a total 810 dynamic timeframes (mean-centered per group, CON and CAN) as nuisance covariates to account for the error variance. To correlate dynamic connectivity measures with subjective craving, nonparametric partial correlation using Spearman’s rho was performed for primary and secondary measures, correcting for the variance of age and average framewise displacement. Multiple comparison corrections were performed using false discovery rate (FDR) of 25% under a restriction that the raw *p*-values are smaller than 0.050 ([Benjamini & Hochberg, 1995](#_ENREF_3)). Alpha level was defined at corrected *p* < 0.050 for all analyses. All the tests were performed using IBM SPSS (IBM Corp. Released 2016. IBM SPSS Statistics for Windows, Version 24.0. Armonk, NY).

# Supplementary Results

## Consistency in Cue-Evoked Craving

A repeated measures ANOVA was run using cue task trial and type as IVs and cue craving scores as DVs. It aimed to evaluate the within-subject effect of cue task trials for the CAN group (*n* = 54). The main effect of trials was not distinct (*F*(4.321, 229.022) = 1.488, *p* = 0.203), but that of types was significant (*F*(1.262, 66.874) = 43.486, *p* < 0.001). A post-hoc pairwise comparison found that the craving upon cannabis cues is higher than neutral and natural reward cues, and the craving upon natural reward cues surpasses that for neutral (Bonferroni corrected *p* < 0.001). This verifies that the during-scan craving was constant across time, and the craving upon cannabis cues was distinguishably the highest for the users. Supplementary Figure 2 shows the course of changes in cue reactivity scores across trials per cue types.

## Group and Task Effects on the Task-Modulated Mean of Dynamic Functional Connectivity

### Connectivity Weight

The task-modulated mean of dynamic connectivity (*d_mean_*) was significantly altered by the task effect, showing *F*(60, 3465) = 12.733, *p* < 0.001. The group effect was not significant with *F*(12, 129) = 1.226, and *p* = 0.272; neither was the group by task interaction that showed *F*(60, 3465) = 0.899, and *p* = 0.695. Supplementary Figure 3 represents the task-modulated *d_mean_* differences of primary measures by task conditions and groups after Bonferroni correction applied.

### Topological Indexes

The effect of task conditions was significant upon task-modulated mean of *G_Eff_*, *G_Mod_* and *G_Res_* on average (*F*(15, 2100) = 77.977, *p* < 0.001). The main effect of group was significant (*F*(3, 138) = 2.850, *p* = 0.040), but individual between-subject effects did not survive after Bonferroni correction (maximum *F*(1,140) = 4.591, minimum uncorrected *p* = 0.034, corrected *p* = 0.102 for *G_Eff_*). The interaction of group and task effects was not significant (*F*(15, 2100) = 1.670, *p* = 0.0503). Supplementary Figure 4 represents task-modulated *d_mean_* differences of secondary measures by task conditions and groups after Bonferroni correction applied.

### Correlation of Subjective Craving with Dynamic Functional Connectivity Measures

For the primary measures, after multiple correction for 144 cases (FDR *q* ≤ 0.250 and uncorrected *p* < 0.050), we found a significant positive correlation of pre-scan craving with WithinCEN except in W2 condition, and a negative correlation with DMN-SN and Amyg-CEN in all conditions. The worst case that passes FDR correction was WithinCEN in C1 condition (uncorrected *p* = 0.028, *q* = 0.237). Supplementary Figure 5 represents the direction and the magnitude of correlation coefficients for *d_mean_* of primary measures and craving scores.

For the secondary measures, after multiple correction for 36 cases, the *d_mean_* of *G_Mod_* showed significant positive correlation with the pre-scan craving for all task conditions. The worst case that passes FDR correction was *G_Mod_* with pre-scan craving in W2 condition (uncorrected *p* = 0.022, *q* = 0.132). Supplementary Figure 6 accounts for the direction and the magnitude of correlation coefficients for *d_mean_* of secondary measures and craving scores.

## Additional Analyses on Non-Smoking Subpopulation

This section aimed to verify if the main effect of group on the task-modulated standard deviation of dynamic FC holds for the non-smoking subpopulation of this study, focusing on NAcc-CEN. All participants were given the question “Since you started regular daily smoking, what is the average number of cigarettes you smoked per day?”, and the answers represented either the daily average number of cigarettes one smokes, or a non-applicable marker showing that one does not regularly smoke. For this additional analysis, participants who did not smoke regularly (non-applicable answers) were included. As a result, 77 for CON, and 27 for CAN were selected.

A repeated measures ANOVA showed that the main effect of task remained significant across six conditions, as in Greenhouse-Geisser *F*(4.605, 460.461) = 15.124 (*p* < 0.001). The main effect of group showed *F*(1, 100) = 4.971 (*p* = 0.028), which is significant, but it will not survive multiple correction with Bonferroni when analyzed for 12 cases as in our main part of the analysis. The nonparametric partial correlation for CAN participants were performed for the pre- (*n* = 26) and during-scan (*n* = 27) craving and the task-modulated standard deviation of dynamic FC in NAcc-CEN. None of the results showed statistical significance (the best case was Spearman’s rho = 0.312, with uncorrected *p* = 0.129 for condition W3). In sum, the main effect of chronic cannabis use upon the variability of dynamic FC selectively remains for NAcc-CEN in the subpopulation. However, the statistical power in general lacks for further conclusions in correlational analyses using subjective craving scores. Supplementary Figure 7 accounts for the task-modulated *d_SD_* differences in NAcc-CEN by task conditions and groups.

# Supplementary Discussion

The average is conceptually a representation for the magnitude of task-modulated functional connectivity that is data-driven, not model-driven as in psychophysiological interaction ([Friston et al., 1997](#_ENREF_6); [McLaren et al., 2012](#_ENREF_11)). The pre-scan or baseline craving showed a positive correlation with the dynamic connectivity average of WithinCEN except the condition of natural reward cue OFF, and a negative correlation with the dynamic connectivity average in DMN-SN and Amyg-CEN. The results with the dynamic connectivity average indicate that it relates to the task-independent baseline of craving, rather than the variability of dynamic connectivity that shows significant correlation with task-modulated subjective craving in NAcc.

The CEN has roles in attentional and inhibitory controls ([Chen et al., 2013](#_ENREF_4); [Sridharan, Levitin, & Menon, 2008](#_ENREF_17)), the impairment of which can be prominent in substance use in accordance to iRISA model ([Goldstein & Volkow, 2011](#_ENREF_8)). The positive correlation of the dynamic connectivity mean (*d_mean_*) within the CEN is consistent with that of the variability (*d_SD_*), presenting the same correlational direction. It may similarly indicate the more active involvement of the CEN in moderating the craving ([Luijten et al., 2014](#_ENREF_10); [Zilverstand, Huang, Alia-Klein, & Goldstein, 2018](#_ENREF_20)), particularly during withdrawal.

The roles of the DMN mainly relate to self-oriented thoughts ([Menon, 2011](#_ENREF_12)), and it can be disrupted by deeper symptoms of addiction ([Moeller & Goldstein, 2014](#_ENREF_13)). The SN, especially the insula, appears to be important in allocating attention towards drug cues ([Naqvi & Bechara, 2010](#_ENREF_14)) that can bias the functional weight between the DMN (internal) and the CEN (external; ([Sutherland, McHugh, Pariyadath, & Stein, 2012](#_ENREF_18)). The negative correlation may therefore indicate that their dynamic dissociation, which will functionally attribute less attention to self-oriented information, can lead to lower pre-scan craving. A previous study found that in the cannabis dependent users, the DMN-SN connectivity shows a positive correlation with the amount of cannabis use per year ([Pujol et al., 2014](#_ENREF_16)), which may indicate their higher attention to cannabis than attention to self.

The dynamic connectivity average in Amyg-CEN had a negative correlation with the pre-scan craving. Amyg is important in appraising the emotional salience of information ([Anderson & Phelps, 2001](#_ENREF_1); [Phan et al., 2004](#_ENREF_15)), and forming reward-associated memories. An optogenetics study showed that stimulation of the central Amyg coupled with a reward signal can induce an addictive behavior ([Tom, Ahuja, Maniates, Freeland, & Robinson, 2019](#_ENREF_19)) and inactivating the same area decreased the withdrawal effect for rats that self-administered nicotine ([Funk et al., 2016](#_ENREF_7)). This suggests that Amyg is implied in denoting the emotional salience of drug cues that leads to further abuse. The FC of the CEN, or dorsolateral prefrontal cortex within the CEN with Amyg, relates to the inhibitory modulation upon emotional distractors, largely for the negative affect ([Anticevic, Barch, & Repovs, 2010](#_ENREF_2); [Dolcos & McCarthy, 2006](#_ENREF_5)). Thus, the negative correlation of the dynamic connectivity average in Amyg-CEN and the abstinence-induced craving may indicate that its momentary increase may be denoting a successful emotional management of craving, which may mitigate the withdrawal-negative affect stage of iRISA model.

# Supplementary Figure Legends

**Supplementary Figure 1**. *Visualization of the intrinsic networks of interest*. Each spheres indicate the geometric centroids of the brain regions mapped on the cortex, as defined in [Gordon et al. (2016)](#_ENREF_9). Three networks are delineated by black (default mode network), blue (central executive network) and red (salience network) colors.

**Supplementary Figure 2**. *Craving for cannabis use upon exposure to neutral, natural reward, and cannabis cues*. Lines represent the raw mean score of craving (minimum 0 to maximum 10) for all cannabis users (CAN, *n* = 54), across 12 trials from two sessions. The shaded area denotes 95% confidence interval across participants.

**Supplementary Figure 3**. *The mean of task-modulated primary measure of dynamic functional connectivity in the healthy control and cannabis users (CON vs. CAN)*. Markers indicate mean of the primary measures per group, and error bars denote the standard error (*n* = 90 for CON, *n* = 54 for CAN). The X-axis represents task conditions (C1, W1, C2, W2, C3, and W3) and Y-axis the magnitude of the present measure. The Y-axis is shared for the same row of three plots. Abbreviations indicate default mode network (DMN), central executive network (CEN), salience network (SN), amygdalae (Amyg) and nuclei accumbens (NAcc). Task conditions abbreviated are neutral cue ON (C1), neutral cue OFF (W1), natural reward cue ON (C2), natural reward cue OFF (W2), cannabis cue ON (C3), cannabis cue OFF (W3). Black circles indicate healthy controls (CON), and red triangles cannabis users (CAN).

**Supplementary Figure 4**. *The mean of task-modulated secondary measure of dynamic functional connectivity in the healthy control and cannabis users (CON vs. CAN)*. Markers indicate mean of the secondary measures per group, and error bars denote the standard error (*n* = 90 for CON, *n* = 54 for CAN). The X-axis represents task conditions (C1, W1, C2, W2, C3, and W3) and Y-axis the magnitude of the present measure. The Y-axis is not shared across the plots. Black circles indicate healthy controls (CON), and red triangles cannabis users (CAN).

**Supplementary Figure 5**. *Correlation of mean of primary measure in dynamic functional connectivity with craving scores in cannabis users (CAN)*. Correlation coefficients that survive the multiple comparison correction using FDR *q* ≤ 0.250 and uncorrected *p* < 0.050 (out of 144 cases) are shown as colored boxes. Each box is color-coded to represent the direction of correlation (Spearman’s rho), where red is positive and blue is negative. The color scale is identical across all types of primary measures. The X-axis represents task conditions (C1, W1, C2, W2, C3, and W3) and Y-axis the craving scores in the order of pre-scan (*n* = 52) and during-scan (*n* = 54). Abbreviations indicate default mode network (DMN), central executive network (CEN), salience network (SN), amygdalae (Amyg) and nuclei accumbens (NAcc). Task conditions abbreviated are neutral cue ON (C1), neutral cue OFF (W1), natural reward cue ON (C2), natural reward cue OFF (W2), cannabis cue ON (C3), cannabis cue OFF (W3).

**Supplementary Figure 6**. *Correlation of mean of secondary measure in dynamic functional connectivity with craving scores in cannabis users (CAN)*. Correlation coefficients that survive the multiple comparison correction using FDR *q* ≤ 0.250 and uncorrected *p* < 0.050 (out of 36 cases) are shown as colored boxes. Each box is color-coded to represent the direction of correlation (Spearman’s rho), where red is positive and blue is negative. The color scale is identical across all types of secondary measures. The X-axis represents task conditions (C1, W1, C2, W2, C3, and W3) and Y-axis the craving scores in the order of pre-scan (*n* = 52) and during-scan (*n* = 54).

**Supplementary Figure 7**. *The standard deviation of task-modulated primary measure of dynamic functional connectivity in the non- or little-smoking subpopulation in healthy control and cannabis users (CON vs. CAN)*. Markers indicate mean of the primary measures per group, and error bars denote the standard error (*n* = 77 for CON, *n* = 27 for CAN). The X-axis represents task conditions (C1, W1, C2, W2, C3, and W3) and Y-axis the magnitude of the standard deviation between central executive network (CEN) and nuclei accumbens (NAcc). Task conditions abbreviated are neutral cue ON (C1), neutral cue OFF (W1), natural reward cue ON (C2), natural reward cue OFF (W2), cannabis cue ON (C3), cannabis cue OFF (W3). The red box indicates the significant main effect of group. Black circles indicate healthy controls (CON), and red triangles cannabis users (CAN).

**References**

Anderson, A. K., & Phelps, E. A. (2001). Lesions of the human amygdala impair enhanced perception of emotionally salient events. *Nature, 411*(6835), 305.

Anticevic, A., Barch, D. M., & Repovs, G. (2010). Resisting emotional interference: brain regions facilitating working memory performance during negative distraction. *Cognitive, Affective, & Behavioral Neuroscience, 10*(2), 159-173.

Benjamini, Y., & Hochberg, Y. (1995). Controlling the false discovery rate: a practical and powerful approach to multiple testing. *Journal of the royal statistical society. Series B (Methodological)*, 289-300.

Chen, A. C., Oathes, D. J., Chang, C., Bradley, T., Zhou, Z.-W., Williams, L. M., . . . Etkin, A. (2013). Causal interactions between fronto-parietal central executive and default-mode networks in humans. *Proceedings of the National Academy of Sciences, 110*(49), 19944-19949.

Dolcos, F., & McCarthy, G. (2006). Brain systems mediating cognitive interference by emotional distraction. *Journal of Neuroscience, 26*(7), 2072-2079. doi:10.1523/JNEUROSCI.5042-05.2006

Friston, K., Buechel, C., Fink, G., Morris, J., Rolls, E., & Dolan, R. J. (1997). Psychophysiological and modulatory interactions in neuroimaging. *Neuroimage, 6*(3), 218-229.

Funk, D., Coen, K., Tamadon, S., Hope, B. T., Shaham, Y., & Le, A. D. (2016). Role of Central Amygdala Neuronal Ensembles in Incubation of Nicotine Craving. *Journal of Neuroscience, 36*(33), 8612-8623. doi:10.1523/JNEUROSCI.1505-16.2016

Goldstein, R. Z., & Volkow, N. D. (2011). Dysfunction of the prefrontal cortex in addiction: neuroimaging findings and clinical implications. *Nature Reviews: Neuroscience, 12*(11), 652-669. doi:10.1038/nrn3119

Gordon, E. M., Laumann, T. O., Adeyemo, B., Huckins, J. F., Kelley, W. M., & Petersen, S. E. (2016). Generation and Evaluation of a Cortical Area Parcellation from Resting-State Correlations. *Cerebral Cortex, 26*(1), 288-303. doi:10.1093/cercor/bhu239

Luijten, M., Machielsen, M. W., Veltman, D. J., Hester, R., de Haan, L., & Franken, I. H. (2014). Systematic review of ERP and fMRI studies investigating inhibitory control and error processing in people with substance dependence and behavioural addictions. *Journal of Psychiatry and Neuroscience, 39*(3), 149-169. doi:10.1503/jpn.130052

McLaren, D. G., Ries, M. L., Xu, G., & Johnson, S. C. (2012). A generalized form of context-dependent psychophysiological interactions (gPPI): a comparison to standard approaches. *Neuroimage, 61*(4), 1277-1286. doi:10.1016/j.neuroimage.2012.03.068

Menon, V. (2011). Large-scale brain networks and psychopathology: a unifying triple network model. *Trends in Cognitive Sciences, 15*(10), 483-506.

Moeller, S. J., & Goldstein, R. Z. (2014). Impaired self-awareness in human addiction: deficient attribution of personal relevance. *Trends in Cognitive Sciences, 18*(12), 635-641. doi:10.1016/j.tics.2014.09.003

Naqvi, N. H., & Bechara, A. (2010). The insula and drug addiction: an interoceptive view of pleasure, urges, and decision-making. *Brain Structure & Function, 214*(5-6), 435-450. doi:10.1007/s00429-010-0268-7

Phan, K. L., Taylor, S. F., Welsh, R. C., Ho, S.-H., Britton, J. C., & Liberzon, I. (2004). Neural correlates of individual ratings of emotional salience: a trial-related fMRI study. *Neuroimage, 21*(2), 768-780.

Pujol, J., Blanco-Hinojo, L., Batalla, A., Lopez-Sola, M., Harrison, B. J., Soriano-Mas, C., . . . Martin-Santos, R. (2014). Functional connectivity alterations in brain networks relevant to self-awareness in chronic cannabis users. *Journal of Psychiatric Research, 51*, 68-78. doi:10.1016/j.jpsychires.2013.12.008

Sridharan, D., Levitin, D. J., & Menon, V. (2008). A critical role for the right fronto-insular cortex in switching between central-executive and default-mode networks. *Proceedings of the National Academy of Sciences, 105*(34), 12569-12574.

Sutherland, M. T., McHugh, M. J., Pariyadath, V., & Stein, E. A. (2012). Resting state functional connectivity in addiction: lessons learned and a road ahead. *Neuroimage, 62*(4), 2281-2295.

Tom, R. L., Ahuja, A., Maniates, H., Freeland, C. M., & Robinson, M. J. (2019). Optogenetic activation of the central amygdala generates addiction‐like preference for reward. *European Journal of Neuroscience, 50*(3), 2086-2100.

Zilverstand, A., Huang, A. S., Alia-Klein, N., & Goldstein, R. Z. (2018). Neuroimaging Impaired Response Inhibition and Salience Attribution in Human Drug Addiction: A Systematic Review. *Neuron, 98*(5), 886-903. doi:10.1016/j.neuron.2018.03.048
