# Supplementary figures and images for: Dynamic functional connectivity between nucleus accumbens and the central executive network relates to chronic cannabis use
Source: Hum Brain Mapp. 2020 May 20;41(13):3637–54. doi: 10.1002/hbm.25036 (PMC7416060; doi:10.1002/hbm.25036)

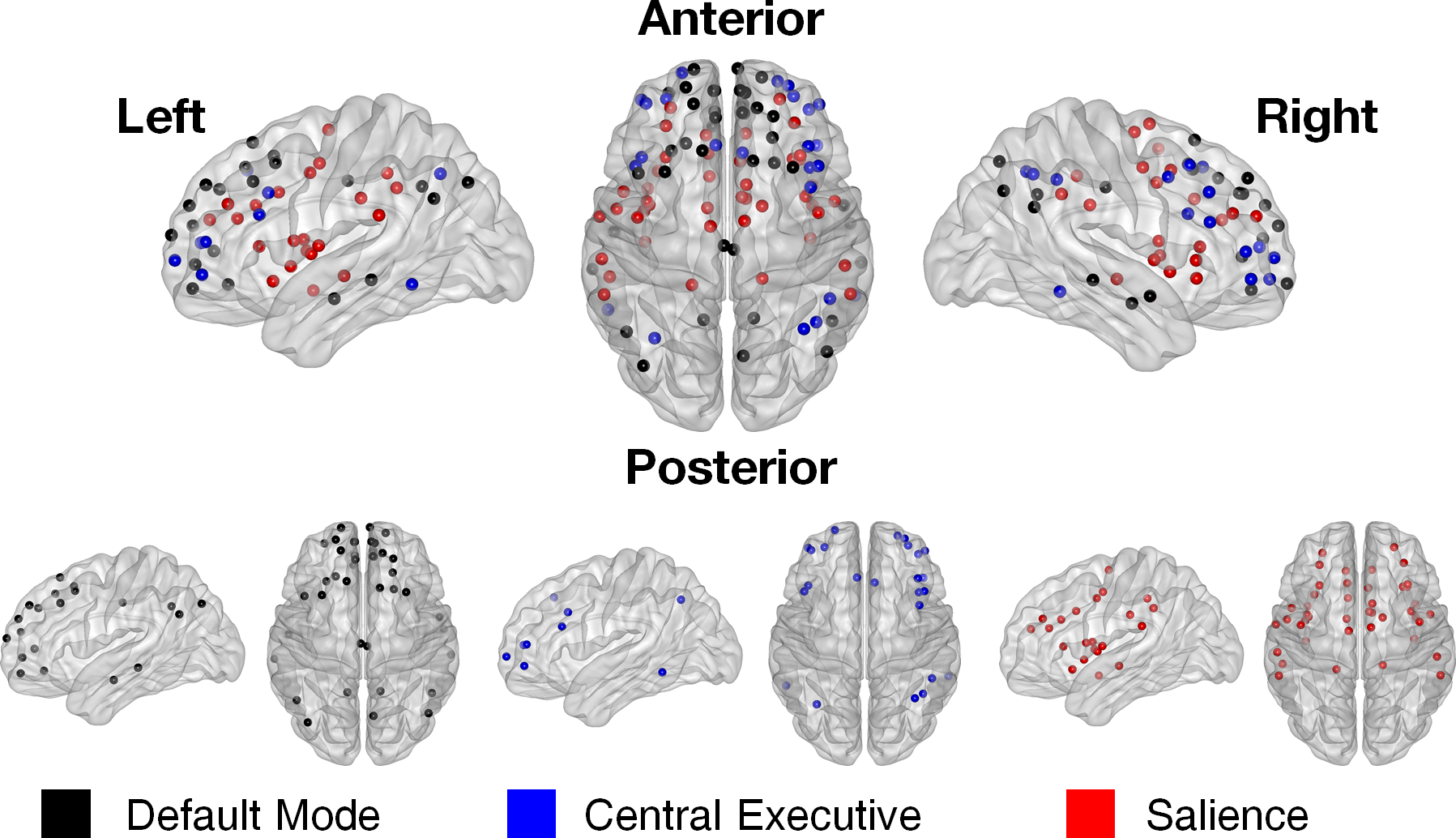

Supplement: Supplementary file 2 — Figure S1 Visualization of the intrinsic networks of interest. Each spheres indicate the geometric centroids of the brain regions mapped on the cortex, as defined in Gordon et al. (2016). Three networks are delineated by black (default mode network), blue (central executive network), and red (salience network) colors. [file HBM-41-3637-s001.png]

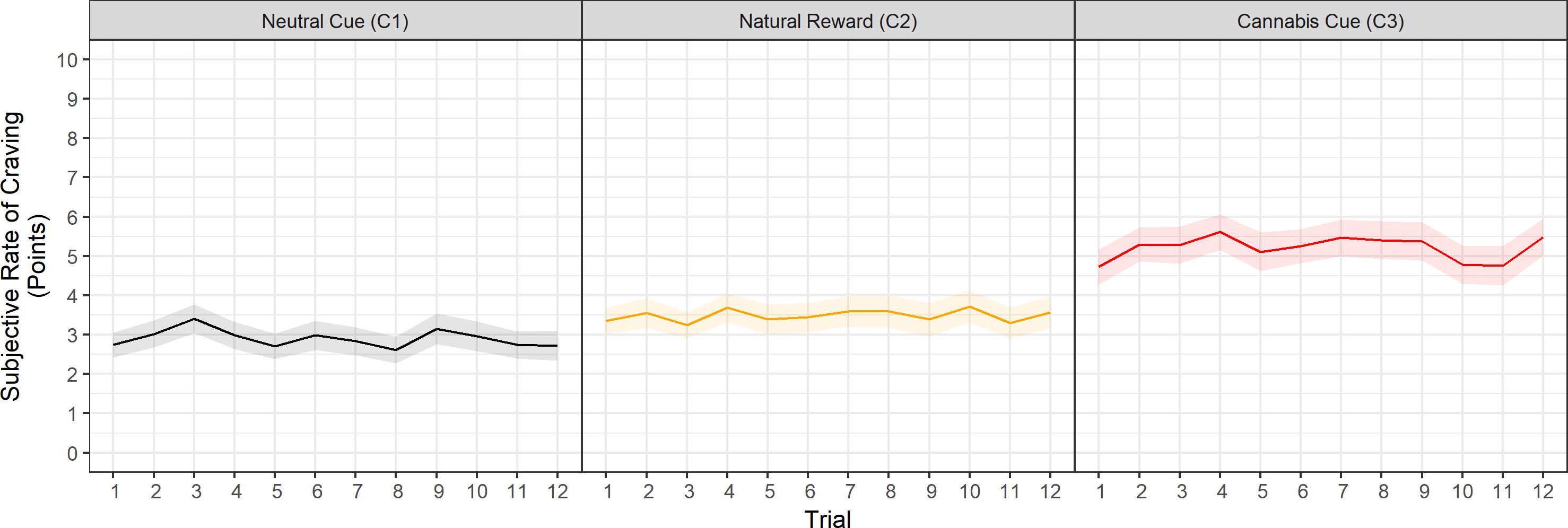

Supplement: Supplementary file 3 — Figure S2 Craving for cannabis use upon exposure to neutral, natural reward, and cannabis cues. Lines represent the raw mean score of craving (minimum 0 to maximum 10) for all cannabis users (CAN, n = 54), across 12 trials from two sessions. The shaded area denotes 95% confidence interval across participants. [file HBM-41-3637-s003.png]

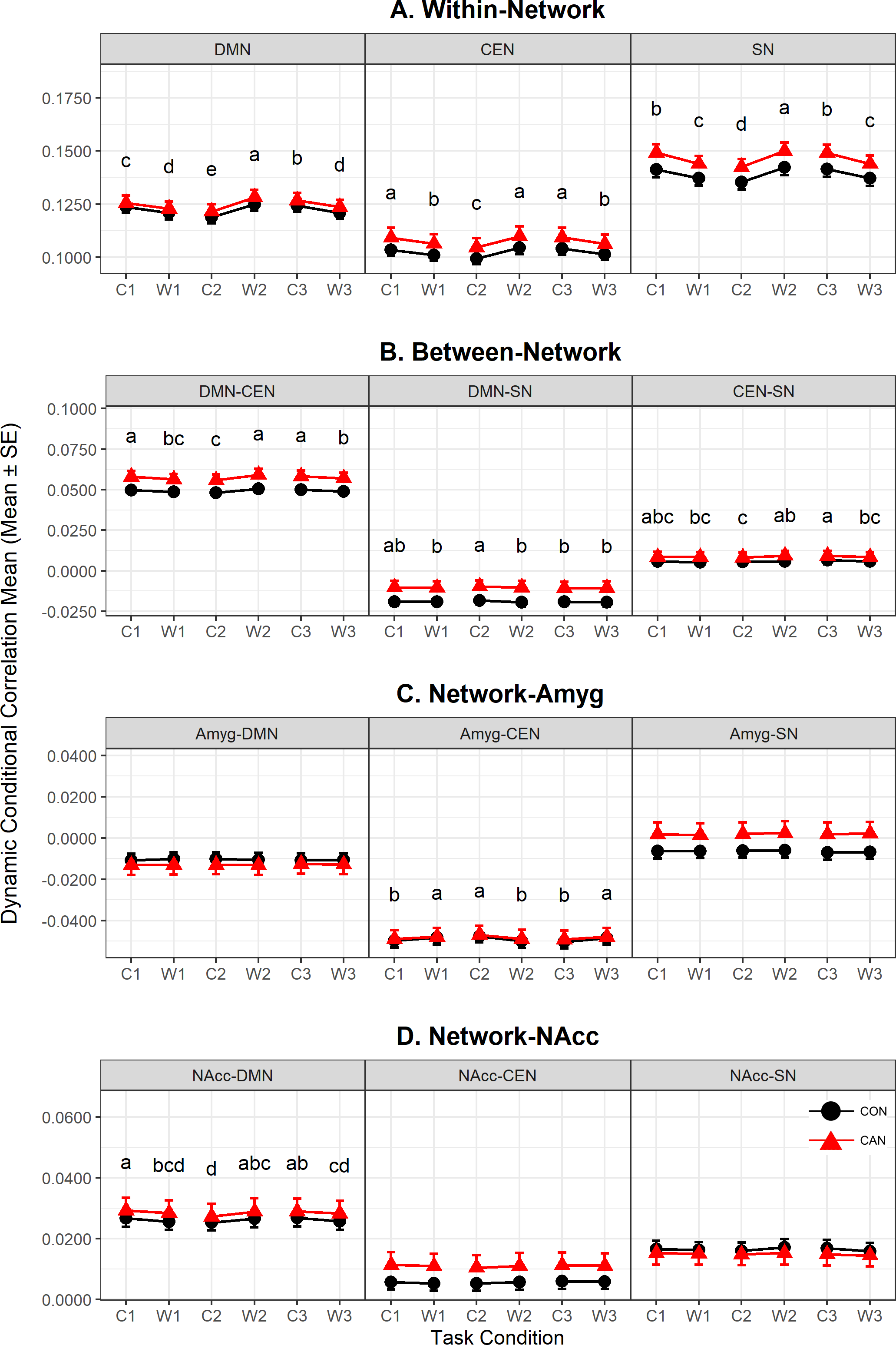

Supplement: Supplementary file 4 — Figure S3 The mean of task‐modulated primary measure of dynamic functional connectivity in the healthy control and cannabis users (CON vs. CAN). Markers indicate mean of the primary measures per group, and error bars denote the standard error (n = 90 for CON, n = 54 for CAN). The X‐axis represents task conditions (C1, W1, C2, W2, C3, and W3) and Y‐axis the magnitude of the present measure. The Y‐axis is shared for the same row of three plots. Abbreviations indicate default mode network (DMN), central executive network (CEN), salience network (SN), amygdalae (Amyg), and nuclei accumbens (NAcc). Task conditions abbreviated are neutral cue ON (C1), neutral cue OFF (W1), natural reward cue ON (C2), natural reward cue OFF (W2), cannabis cue ON (C3), cannabis cue OFF (W3). Black circles indicate healthy controls (CON), and red triangles cannabis users (CAN). [file HBM-41-3637-s004.png]

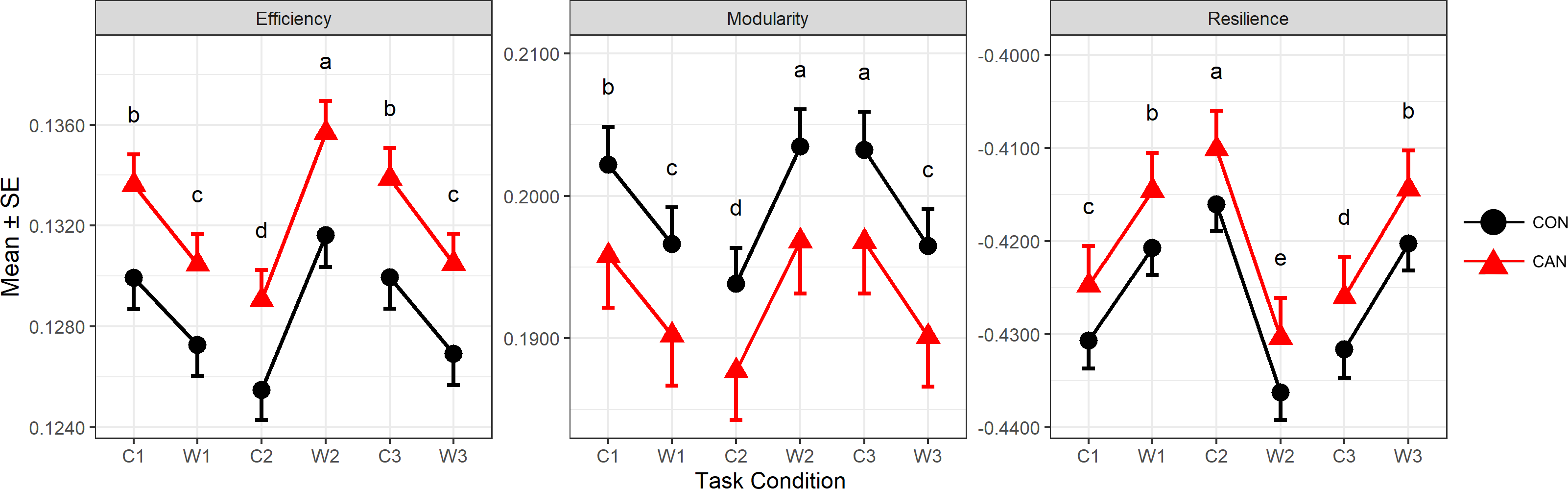

Supplement: Supplementary file 5 — Figure S4 The mean of task‐modulated secondary measure of dynamic functional connectivity in the healthy control and cannabis users (CON vs. CAN). Markers indicate mean of the secondary measures per group, and error bars denote the standard error (n = 90 for CON, n = 54 for CAN). The X‐axis represents task conditions (C1, W1, C2, W2, C3, and W3) and Y‐axis the magnitude of the present measure. The Y‐axis is not shared across the plots. Black circles indicate healthy controls (CON), and red triangles cannabis users (CAN). [file HBM-41-3637-s005.png]

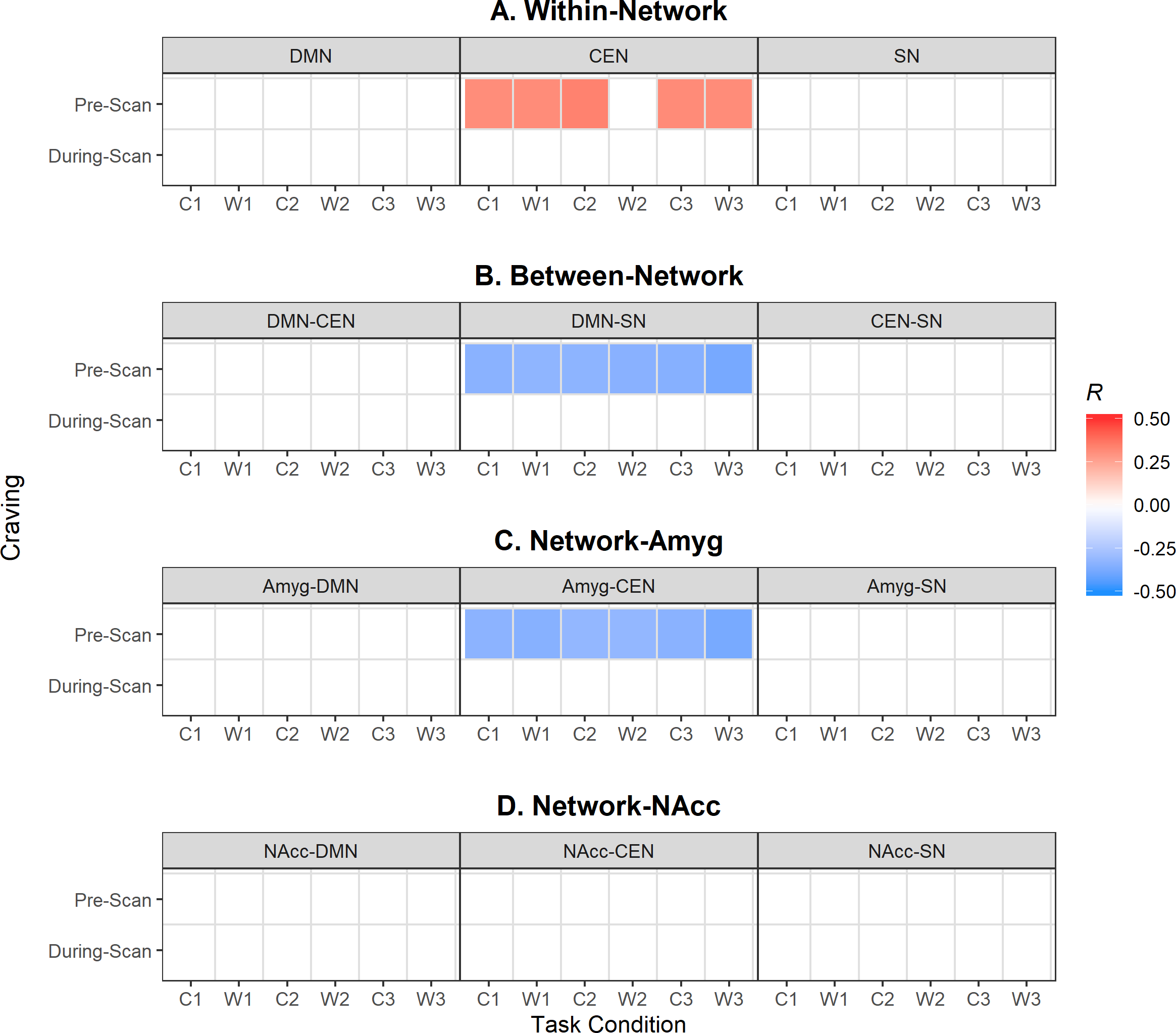

Supplement: Supplementary file 6 — Figure S5 Correlation of mean of primary measure in dynamic functional connectivity with craving scores in cannabis users (CAN). Correlation coefficients that survive the multiple comparison correction using FDR q ≤ 0.250 and uncorrected p < .050 (out of 144 cases) are shown as colored boxes. Each box is color‐coded to represent the direction of correlation (Spearman's rho), where red is positive and blue is negative. The color scale is identical across all types of primary measures. The X‐axis represents task conditions (C1, W1, C2, W2, C3, and W3) and Y‐axis the craving scores in the order of prescan (n = 52) and during‐scan (n = 54). Abbreviations indicate default mode network (DMN), central executive network (CEN), salience network (SN), amygdalae (Amyg), and nuclei accumbens (NAcc). Task conditions abbreviated are neutral cue ON (C1), neutral cue OFF (W1), natural reward cue ON (C2), natural reward cue OFF (W2), cannabis cue ON (C3), cannabis cue OFF (W3). [file HBM-41-3637-s006.png]

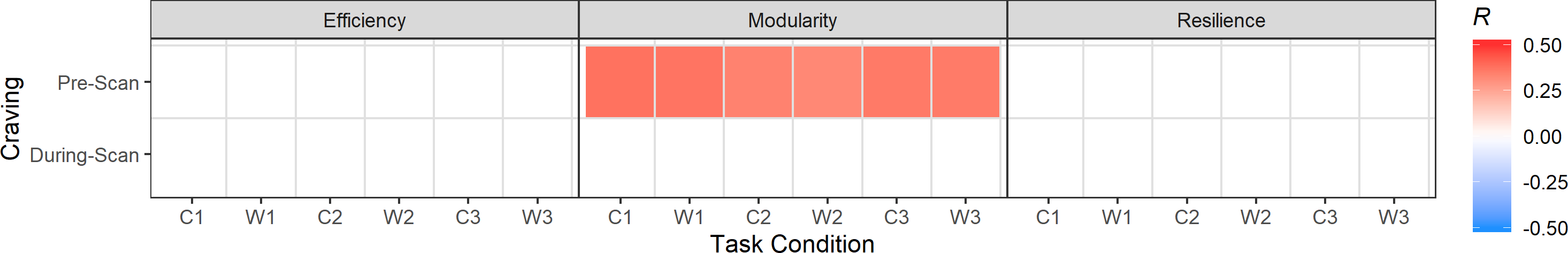

Supplement: Supplementary file 7 — FIGURE S6 Correlation of mean of secondary measure in dynamic functional connectivity with craving scores in cannabis users (CAN). Correlation coefficients that survive the multiple comparison correction using FDR q ≤ 0.250 and uncorrected p < .050 (out of 36 cases) are shown as colored boxes. Each box is color‐coded to represent the direction of correlation (Spearman's rho), where red is positive and blue is negative. The color scale is identical across all types of secondary measures. The X‐axis represents task conditions (C1, W1, C2, W2, C3, and W3) and Y‐axis the craving scores in the order of prescan (n = 52) and during‐scan (n = 54). [file HBM-41-3637-s007.png]

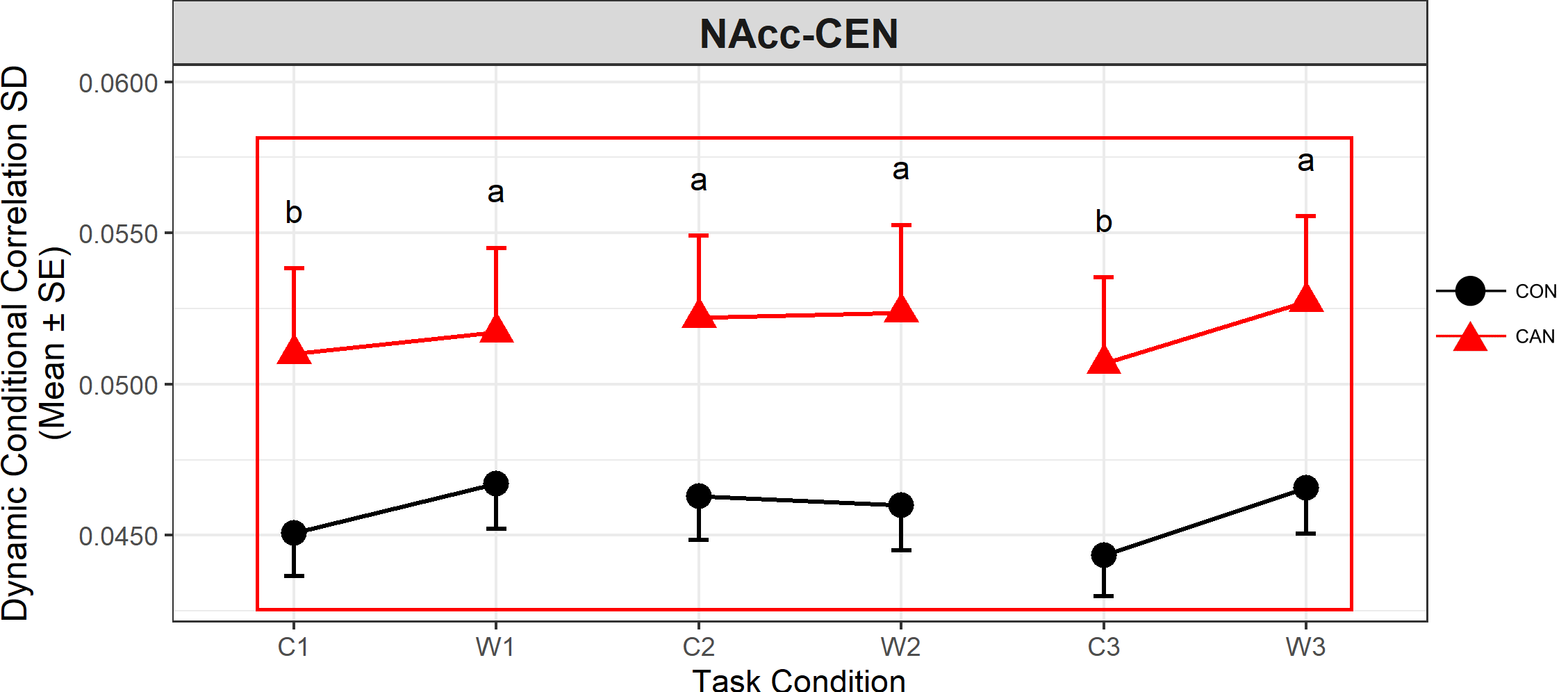

Supplement: Supplementary file 8 — Figure S7 The standard deviation of task‐modulated primary measure of dynamic functional connectivity in the non‐ or little‐smoking subpopulation in healthy control and cannabis users (CON vs. CAN). Markers indicate mean of the primary measures per group, and error bars denote the standard error (n = 77 for CON, n = 27 for CAN). The X‐axis represents task conditions (C1, W1, C2, W2, C3, and W3) and Y‐axis the magnitude of the standard deviation between central executive network (CEN) and nuclei accumbens (NAcc). Task conditions abbreviated are neutral cue ON (C1), neutral cue OFF (W1), natural reward cue ON (C2), natural reward cue OFF (W2), cannabis cue ON (C3), cannabis cue OFF (W3). The red box indicates the significant main effect of group. Black circles indicate healthy controls (CON), and red triangles cannabis users (CAN). [file HBM-41-3637-s008.png]
